# Ratiometric Electrochemistry: Improving the Robustness, Reproducibility and Reliability of Biosensors

**DOI:** 10.3390/molecules26082130

**Published:** 2021-04-07

**Authors:** Sam A. Spring, Sean Goggins, Christopher G. Frost

**Affiliations:** 1 Department of Chemistry, University of Bath, Claverton Down, Bath BA2 7AY, UK; Sam.Spring@warwick.ac.uk; 2 Bio-Techne (Tocris), The Watkins Building, Atlantic Road, Avonmouth, Bristol BS11 9QD, UK; sean.goggins@bio-techne.com

**Keywords:** electrochemical biosensors, ratiometric detection, secondary redox-active labelling, chemodosimeters, dual-channel systems

## Abstract

Electrochemical biosensors are an increasingly attractive option for the development of a novel analyte detection method, especially when integration within a point-of-use device is the overall objective. In this context, accuracy and sensitivity are not compromised when working with opaque samples as the electrical readout signal can be directly read by a device without the need for any signal transduction. However, electrochemical detection can be susceptible to substantial signal drift and increased signal error. This is most apparent when analysing complex mixtures and when using small, single-use, screen-printed electrodes. Over recent years, analytical scientists have taken inspiration from self-referencing ratiometric fluorescence methods to counteract these problems and have begun to develop ratiometric electrochemical protocols to improve sensor accuracy and reliability. This review will provide coverage of key developments in ratiometric electrochemical (bio)sensors, highlighting innovative assay design, and the experiments performed that challenge assay robustness and reliability.

## 1. Introduction

The development of electrochemical biosensors for applications in point-of-care devices is appealing and growing in popularity. Principally, this is due to the ability to obtain good sensitivity at very low cost, the potential for multiplexing [1,2,3] and the capability for miniaturisation [4] and sample-to-signal requiring little-to-no user manipulation. Despite their rapid development over the last few years, there has been significant confusion in the literature that ratiometric detection offers increased signal intensity, and improved assay sensitivity in comparison to traditional ‘switch-on’ detection methods [5,6,7]. Improved sensitivity greater than an order of magnitude is typically achieved through rigorous assay development and a thorough investigation of the assay parameters including the mechanism of the analyte–probe recognition event; the electrode composition and its surface area; the electrochemical experiment employed; composition of the buffer, concentration and pH; and if any signal amplification protocols are used [8,9]. If an analyte detection assay is maintained with these assay parameters kept identical, but switches the detection method from ‘switch-on’ with a single redox-active compound to a ratiometric detection method with two redox active compounds, then the sensitivity of the assay should remain similar. Indeed, one of the first published examples of a ratiometric electrochemical biosensor reported a limit of detection (LOD) of 1.9 nM for the sum of the current difference using two redox-active labels, whereas LODs for the labels individually were 5.2 and 4.8 nM, respectively [10].

The true benefit of employing a ratiometric detection method is not for sensitivity, but rather for improved assay reliability and reproducibility. This overall increase in assay accuracy should provide the analyst with greater confidence in the analyte concentration, which becomes increasingly important when moving analyte testing out of clean laboratories with controlled environments, and into the field. Here, a host of external environmental factors can all lead to significant disparities in signal intensity, such as temperature, humidity, sample volume, electrode surface area, out-of-calibration instrumentation, and contamination. The accumulated errors associated with these factors can be minimised by including a redox-active internal standard into the design of the electrochemical sensor. In the case of a positive scenario, this allows for the signal produced to be referenced against the internal standard, and analyte concentration can therefore be accurately determined by the ratio between these two signals. Variations in signal intensity caused by any external factors, and not by the concentration of the target, should affect both redox-active labels equally and would therefore be cancelled out when calculating the analyte concentration from the ratio of the two peaks. As such, dual-signalling assays, where both signals are attributed to the same redox-active label, do not offer the same unique self-correcting properties, and are not considered ratiometric [11].

**Figure 1:**
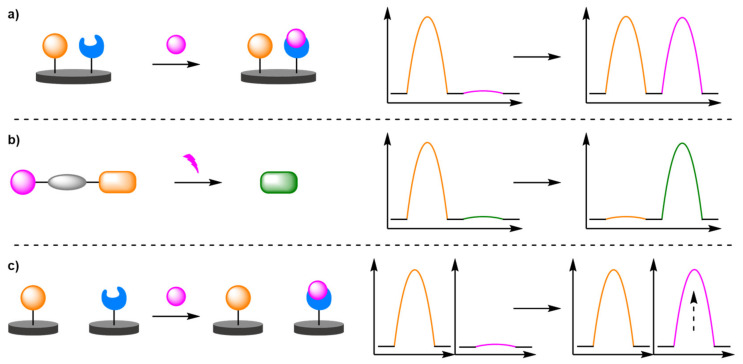
Overview of ratiometric electrochemical biosensors: (**a**) secondary redox-active labelling; (**b**) chemodosimeters; (**c**) dual-channel systems.

The review is divided into three main sections according to the approaches that have been typically deployed to date. Firstly, through an internal standard as a secondary redox-active label bound to a probe or directly to the electrode or unbound in the assay; secondly through a ratiometric electrochemical chemodosimeter that undergoes a selective shift in electrochemical signal; and finally, a dual-channel system that employs two working electrodes (Figure 1).

## 2. Secondary Redox-Active Labelling

The most common approach to achieving ratiometric electrochemical sensing is to use a second redox-active label with a distinct redox potential (E_ox_), in addition to the redox-active label used to indicate the presence of the target. Depending on the target type, there are three distinct protocols in which to achieve this. For DNA, secondary labelling of the probe, or other DNA architecture, which can maintain proximity to the electrode to afford an internal reference is often employed. For other analytes, including metal ions, small molecules, and proteins, direct modification of the electrode with a redox-active internal reference is typical. A less prominent strategy introduces an unbound electroactive reference into the assay for the detection of analytes with unmodified electrodes.

### 2.1. Secondary Labelling of Solid-Supported DNA Structures

The dual-labelled DNA probe approach to ratiometric electrochemical sensing is a general one as many articles based upon this concept have since been published, often employing ferrocene (Fc) and methylene blue (MB) as redox labels due to their facile and clearly distinguishable oxidation potentials. Some have aimed to improve the sensitivity or selectivity of the DNA detection assay, while others have utilised the versatility and excellent selectivity of DNA aptamers to extend the method to the detection of other analytes of interest.

**Figure 2:**
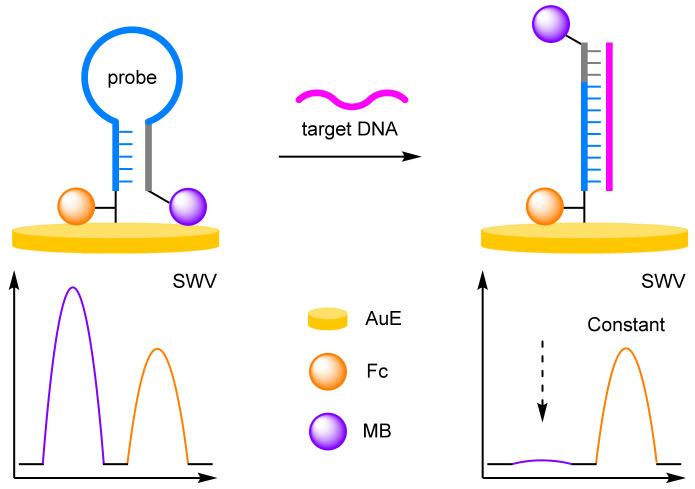
Schematic representation of a biosensor for target DNA detection.

Sessler and Ellington were the first to demonstrate the improved robustness and reliability that can be achieved when using a ratiometric electrochemical detection method (Figure 2) [12]. Building upon the reagentless ‘switch-off’ electrochemical DNA detection protocol developed by Plaxco [13], the groups synthesised a 37 mer DNA probe, which was labelled at the 3′ end with a Fc derivative (3′-Fc), and at the 5′ end with MB (5′-MB). The probe was immobilised onto a gold electrode (AuE) via a thiol linkage at its 3′ end. In the absence of the target DNA, the probe adopts a molecular beacon conformation, which places both redox-active labels in proximity to the electrode. Square-wave voltammetry (SWV) curves showed that both electrochemical labels have distinct E_ox_ (*I_Fc_* 440 mV, *I_MB_* −265 mV), a crucial criterium for achieving ratiometric electrochemical detection. The reproducibility was then rigorously tested by measuring the background current 50 times across eight different electrodes over multiple different days. Using this approach, the normalised current ((*I_MB_*/*I_Fc_*)^0^) obtained with the ratiometric method showed a significantly lower variance in comparison to the single-labelled method. Upon hybridisation with the target DNA, a T-lymphotropic virus type I gene, the probe undergoes a conformational change which results in the MB redox label being moved away from the electrode. As such, the current measured for the Fc label remains similar to the background, while the current measured for the MB label decreases with increasing target concentration. The LOD was calculated to be 25 pM, comparable to the single-redox label approach. More importantly, the ratiometric method was found to be far more reliable with a correlation coefficient calculated to be 0.997, in comparison to 0.958 for the ‘switch-off’ approach.

#### 2.1.1. Binding Modes

The utility of DNA-based biosensors has been expanded past direct detection of target DNA. Inspired by non-ratiometric examples, DNA-based biosensors have been developed for the detection of heavy metal ions and the direct detection of enzymes. Antibodies and aptamers have become common in biosensors [14,15,16], and their incorporation into ratiometric electrochemical biosensors further expanded the scope of the detectable analytes to include proteins and other small molecules of interest.

(a) Heavy Metals

**Figure 3:**
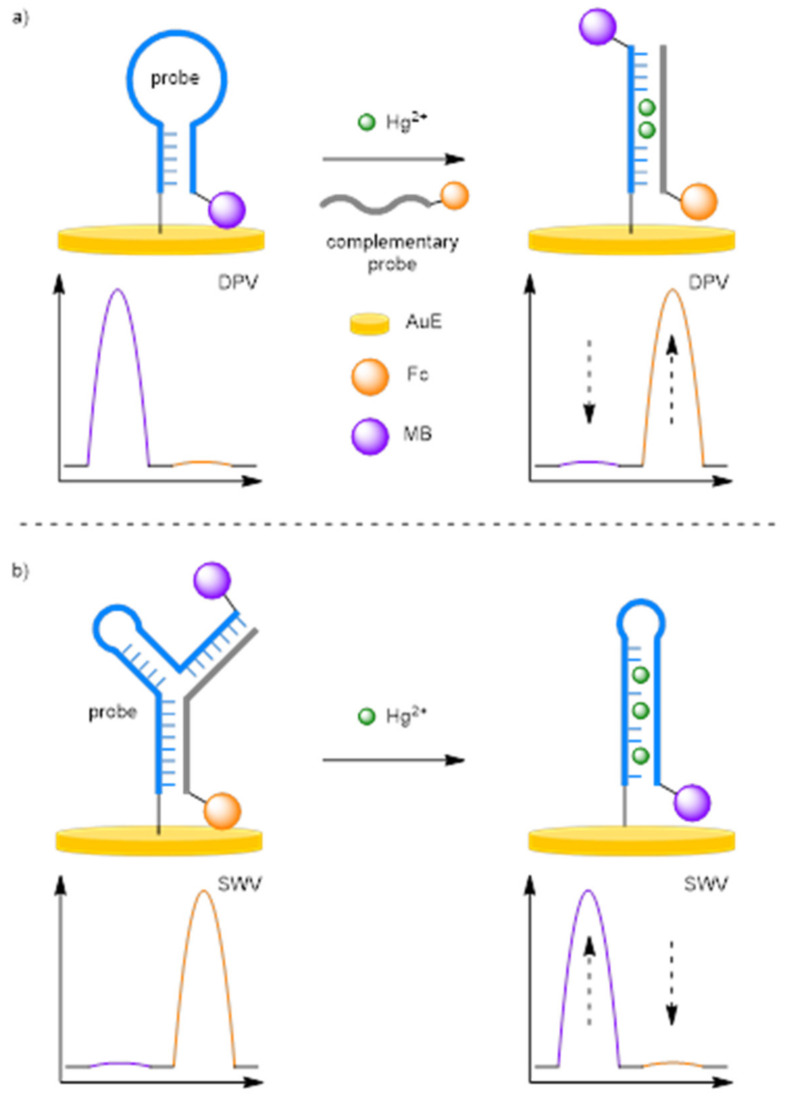
(**a**) The detection of Hg^2+^ using DNA-templated metallisation of a thymine-rich hairpin DNA probe. (**b**) The detection of Hg^2+^ using DNA-templated metallisation of a Y-shaped probe.

Due to the strong binding affinity of DNA to heavy metals, and through an emerging technique known as DNA-templated metallisation [17], DNA can be used as an analyte-recognition element in assays for the detection of heavy metals and by extension, small molecules. Zhang and Chen et al. were the first to demonstrate that a ratiometric electrochemical endpoint detection method could be applied to such a heavy metal detection assay, through exploiting the high binding affinity between mercury ions and thymine nucleobases (Figure 3a) [18]. A thymine-rich hairpin DNA probe strand was solid supported onto a AuE via a thiol linkage at its 5′ end and 3′-MB labelled. In the absence of the target, the hairpin loop remains in its closed conformer and current for MB was observed via differential pulse voltammetry (DPV) due its proximity to the electrode. In the presence of Hg^2+^, mercury-mediated binding between the solid-supported probe strand and the complementary 3′-Fc-labelled signal probe occurs, forming a double-stranded duplex. The rigid structure moves the MB label away and the Fc label towards the electrode. A positive assay sees a decrease in current for MB, and an increase in current for Fc. The assay showed a dynamic range between 5 μM and 0.5 nM, a correlation coefficient of 0.997, and an LOD of 0.08 nM. The reproducibility of the assay was only through five repeated experiments on different electrodes which achieved a relative standard deviation (RSD) of 3.7%.

Luo and Li et al. developed a similar ratiometric electrochemical mercury detection assay (Figure 3b) [19]. In this instance, a Y-shaped probe was formed from the two DNA stands, with a Fc label close to the electrode, and an MB label away from the electrode. The probe, in the presence of mercury ions, would disassemble to release the Fc-labelled strand into solution, and allowing the solid-supported DNA to reform the hairpin conformer. Nearly identical numbers were obtained for this assay: a dynamic range of 5 μM–1 nM, an LOD of 0.09 nM, a correlation coefficient of 0.995, and an RSD of 3.6% when five individually prepared electrodes were tested at 1 nM Hg^2+^.

(b) Antibodies

**Figure 4:**
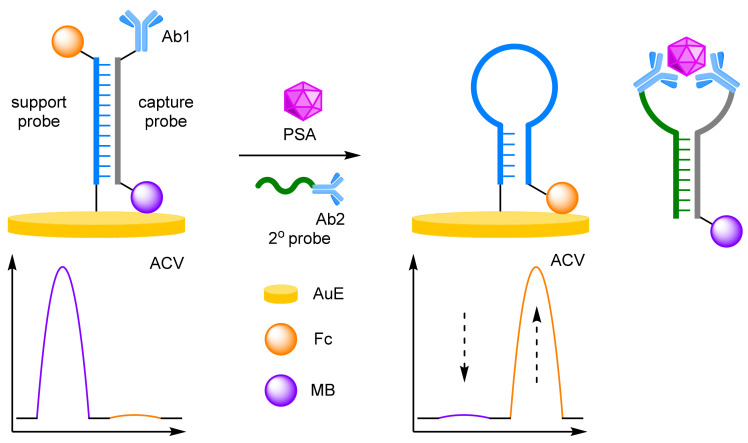
Schematic representation of an antibody-based biosensor for the detection of protein-specific antigen (PSA).

The effective conjugation of DNA to other biomolecules such as antibodies, has allowed for the construction of DNA-immobilised, sandwich-type immunoassays for efficient protein detection. As DNA is the backbone to these assays, ratiometric electrochemical detection methods can also be applied to improve assay reliability and robustness. Ju et al. in particular have pioneered this approach, utilising ratiometric electrochemistry in concert with an immunoassay for the detection of prostate-specific antigen (PSA) (Figure 4) [20]. A hairpin support probe was labelled 5′-Fc and immobilised at its 3′ end to a AuE, and hybridised with a complementary capture probe labelled 5′-MB and its 3′ end with a secondary antibody. In the absence of the target cancer biomarker, the double-stranded DNA structure keeps the Fc label away from the electrode, while the MB label is close. In a positive assay, an immunoassay-type sandwich structure forms, with the assistance of another single-stranded DNA probe bearing a PSA-specific antibody. This secondary probe, complementary to that of the capture probe, displaces the support probe and releases the sandwich structure into solution. The support probe reverts to its preferred hairpin structure, which places the Fc label close to the electrode. Target PSA concentration can then be determined by the ratio between the two peak currents observed by alternating current voltammetry (ACV). A successful sandwich immunoassay structure is a prerequisite for strand displacement, as without the target, no change in current is observed for either redox label. Again, the excellent robustness of the ratiometric detection method was exemplified, with a correlation coefficient of 0.999 over a target concentration range of 0.05–100 ng mL^−1^, along with an LOD of 16 pg mL^−1^. The same group also demonstrated that the assay can be performed in reverse, such that the hairpin support probe could be opened by the presence of an analyte-initialised secondary DNA structure, which greatly reduced total assay time [21]. PSA could be detected over a similar concentration range, between 0.01 and 200 nM, with a correlation coefficient of 0.997, and an LOD of 4.3 pg mL^−1^ after just a 30 min incubation.

(c) Aptamers

DNA aptamers have recently emerged as a tuneable, synthetic alternative to antibodies as a way to selectively bind to a target analyte other than DNA [22,23,24]. Coupled with their versatility and robustness when applied to solid-supported electrochemical biosensors, DNA aptamers have been utilised in combination with ratiometric electrochemistry to achieve selective and accurate analyte detection. This extends the use of DNA within biosensors from solely DNA detection to other biomolecules such as proteins and liposaccharides, as well as small molecules such as plastic contaminants and drugs.

**Figure 5:**
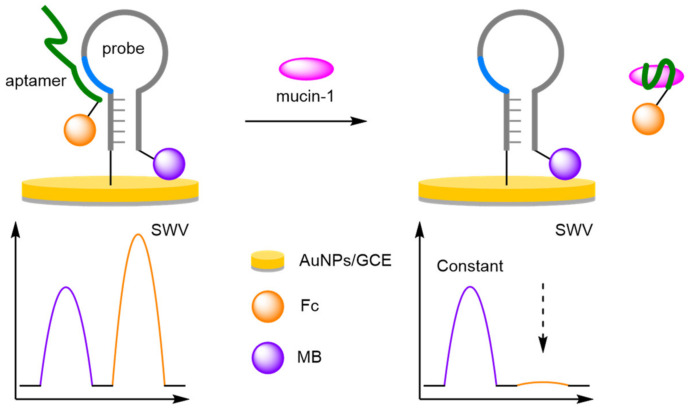
Schematic representation of an aptamer-based biosensor for the detection of mucin-1.

For the electrochemical detection of tumour biomarkers, Xiang et al. showed that a DNA aptamer, selective for mucin-1, could be combined with a ‘switch-off’ ratiometric method for improved robustness and reliability (Figure 5) [25]. To achieve this, a hairpin reference probe labelled 3′-MB was anchored to a gold nanoparticle-coated glassy carbon electrode (AuNPs/GCE), through a thiol linkage at its 5′ end. Close to the 5′ end, but within the loop itself, an eleven base-pair link sequence complementary to that of a target-binding 3′-Fc-labelled aptamer was designed into the probe. In the absence of the target, hybridisation between the probe and the aptamer results in both redox-active labels being placed in proximity to the electrode, with two current peaks observed using SWV. In the presence of mucin-1, competitive binding for the aptamer occurs. This results in disassociation from the probe, and removal of the Fc label. Over a concentration range between 1 nM and 1 μM of mucin-1 in phosphate buffer solution (PBS), a correlation coefficient of 0.996 was observed and an LOD of 0.83 nM calculated. More importantly, the excellent reproducibility of the ratiometric detection method was also demonstrated with 30 experiments performed over 10 electrodes. The RSD of the background and positive response was found to be 4.0% and 5.2%, respectively, which compares favourably with the values for the non-ratiometric method of 15.8% and 17.1%.

**Figure 6:**
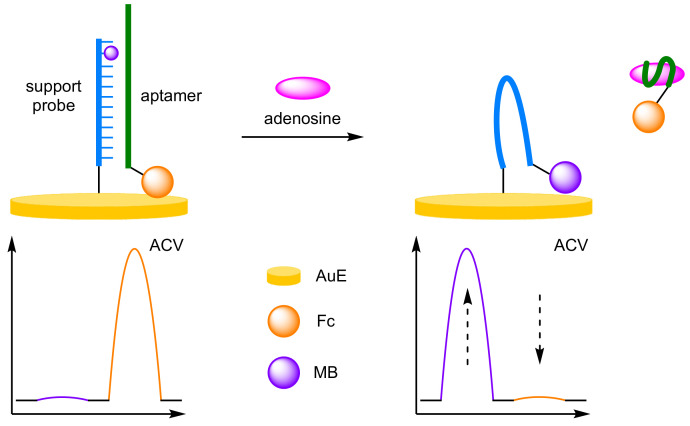
Schematic representation of an aptamer-based biosensor for the detection of adenosine.

The specificity of aptamers towards free nucleotides was utilised by Zhang et al. for the detection of adenosine [26]. They exploited the strong binding affinity of MB-modified thymine residues towards alternating AT base sequences, resulting in a ratiometric ‘switch-on–switch-off’ biosensor (Figure 6). A 5′-thiolated DNA support probe, containing the MB-modified thymine residue penultimate to the 3′ end, was immobilised onto a AuE. The 3′-Fc-labelled aptamer formed a stable duplex with the support probe, encapsulating the MB label, and placing the Fc label close to the electrode. On addition of adenosine, aptamer binding induces disassociations of the duplex, removing the Fc label. The immobilised strand brings the MB label close to the surface, with a resultant current increase at −280 mV (*I_MB_*) and concurrent decrease at 390 mV (*I_Fc_*). Quantification of the ratiometric currents via ACV could then be used to determine adenosine concentration over a dynamic range of 0.1 nM–10 µM, with a correlation coefficient of 0.998, an order of magnitude larger than the single label ‘switch-on’ or ‘switch-off’ assays. The biosensor demonstrated good reproducibility at 100 nM concentrations, and the regeneration was possible on incubation with the labelled aptamer to reform the duplex, with an RSD of 4.8% reported after 5 cycles.

**Figure 7:**
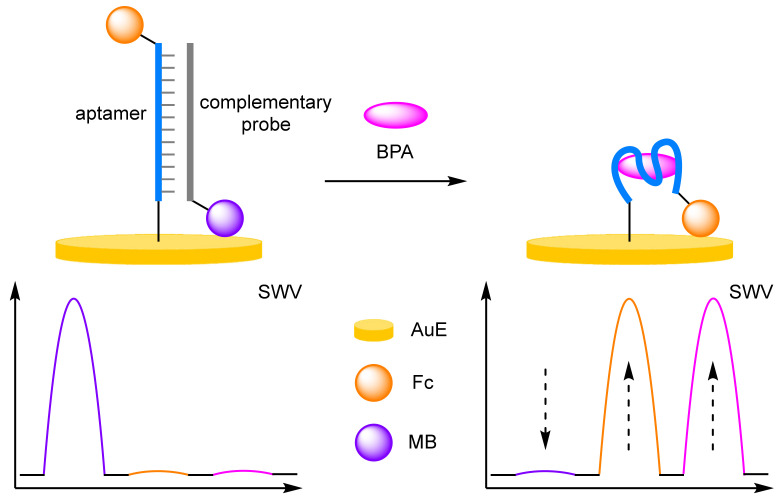
Schematic representation of an aptamer-based biosensor for the detection of bisphenol A (BPA) utilising the redox activity of BPA.

When target compounds themselves have their own facile E_ox_, one way to avoid peak overlap is to choose a reference label and a reporter label with sufficiently different E_ox_ to that of the target. Zhang and Chen et al. deliberately incorporated the E_ox_ of bisphenol-A (BPA) into their assay design (Figure 7) [27]. In this triple-signalling assay, a double-stranded duplex was solid supported onto a AuE containing an immobilised BPA-specific aptamer labelled 3′-Fc, and hybridised with a complementary strand labelled 3′-MB. In the presence of BPA, displacement of the complementary strand with BPA occurs with removal of the MB label. The target is selectively detected by SWV through the reduction in the peak at −280 mV (*I_MB_*), as well as the concomitant increase in both peaks at 280 mV (*I_Fc_*) at ≈575 mV (*I_BPA_*). By taking the sum of the current changes for all redox active species (*ΔI_Fc_* + |*ΔI_MB_*| + *ΔI_BPA_*), the LOD for the assay was calculated to be 0.19 pM. Although lower than taking the current from any one of the redox labels individually, this is still within the same order of magnitude as a single label assay, and a structurally similar biosensor that utilised a single redox label on the complementary strand [28]. This further reinforces the notion that increasing the number of redox-active labels does not afford great leaps in sensitivity.

**Figure 8:**
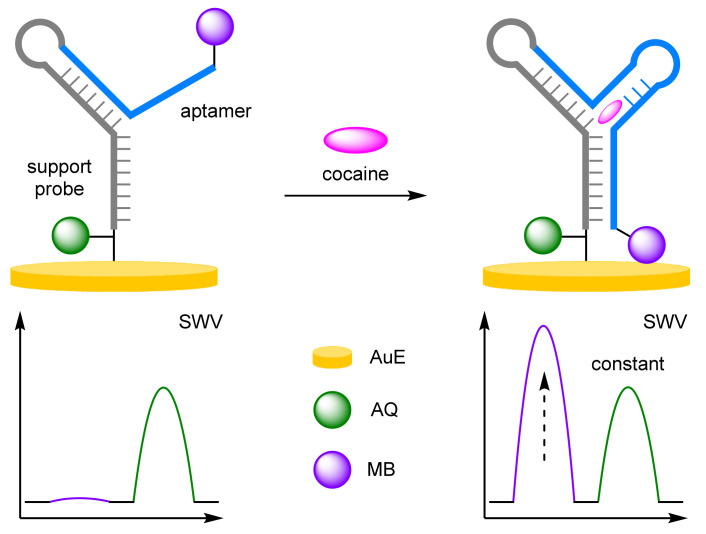
Schematic representation of an aptamer-based biosensor for the detection of cocaine in whole blood.

To challenge the ratiometric method further than buffered solutions, Plaxco et al. developed an electrochemical DNA aptamer-based sensor for the monitoring of cocaine in undiluted whole blood (Figure 8) [29]. Whole blood is one of the most challenging mediums in which electrochemical sensors can be deployed due to the large number of potential contaminants present in blood that could cause non-specific binding and electrode fouling, leading to severe drift of the baseline current. To circumvent this issue, a DNA aptamer specific towards cocaine, was immobilised onto a AuE and was labelled at the end proximal to the electrode with anthraquinone (AQ) and labelled at the distal end with MB. The redox labels employed have significantly distinguishable E_ox_ (*I_AQ_* −420 mV, *I_MB_* −260 mV, vs. Ag/AgCl), both are stable, and both have similar physical properties, making them ideal for ratiometric electrochemical sensing. The current observed for the AQ reference label was shown to be largely insensitive to the target, whereas the current observed for the MB signal label showed concentration-dependent increases, thus demonstrating the feasibility of the method. Continuous monitoring for 15 hours in whole blood in the absence of the target was found to significantly reduce baseline drift from as much as 50% to less than 5%. This allowed greater biosensor accuracy when reporting target concentrations of 0.2 and 1 mM, which could easily be determined at any timepoint within several hours of continuous blood monitoring. By simply switching the DNA aptamer used, this general approach could also be applied to the detection of other small molecule drugs such as kanamycin and doxorubicin. However, the latter was found to have a similar E_ox_ to that of AQ, exposing an unfortunate limitation of electrochemical sensing.

(d) Enzyme Detection

**Figure 9:**
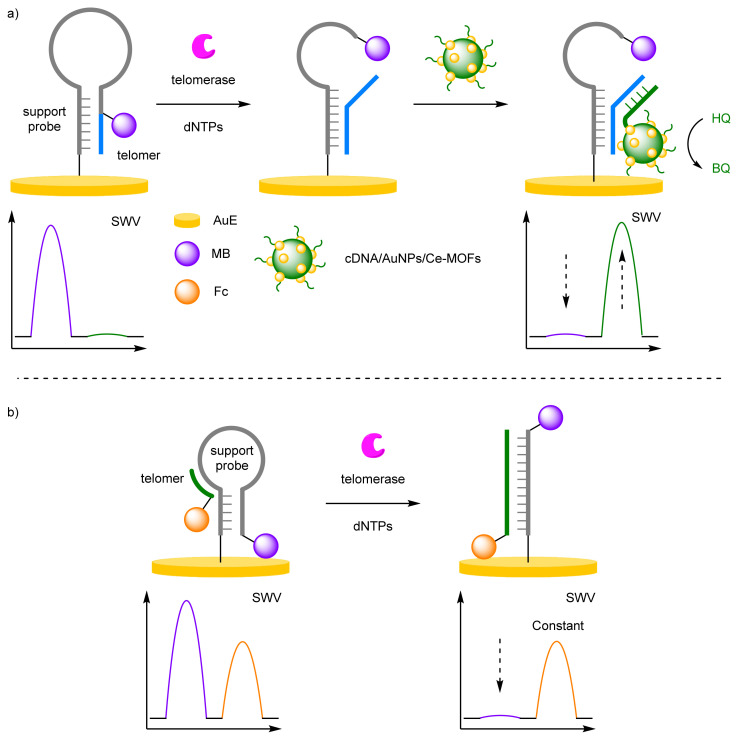
Schematic representation of two biosensor for the detection of telomerase: (**a**) a MOF based approach; (**b**) a hybridisation approach.

When the analyte of interest is an enzyme, biosensors can be designed to utilise their in-built catalytic activity. For example, telomerase adds repeat units to the 3′ end of telomeres and are over expressed in cancer cells. Their chain elongation properties have been incorporated into biosensor design for the facile detection of cancer cells. Lei et al. utilised cerium metal organic frameworks (Ce-MOFs), labelled with AuNPs and capture DNA to detect telomerase activity (Figure 9a) [30]. An MB-labelled hairpin was immobilised onto a AuE, with a hybridised telomer proximal to the electrode, and the hairpin conformation bringing the MB label close to the electrode surface. In the presence of telomerase and deoxyribonucleotides (dNTPs), chain extension elongates the primer disrupting the hairpin conformer, removing the MB label from the surface. The capture DNA is complementary to the elongated strand, which brings the CeMOF structure close to the electrode. The MOF catalyses the conversion of hydroquinone (HQ) to benzoquinone (BQ), which possess a distinct E_ox_ at 280 mV, allowing for ratiometric electrochemical analysis. A dynamic range was reported of 2 × 10^2^ to 2 × 10^6^ HeLa cell mL^−1^ with a calculated LOD of 27 HeLa cell mL^−1^.

A biosensor developed by Miao et al. used a simpler strategy for the detection of telomerase (Figure 9b) [31]. A 5′-MB-labelled strand was immobilised onto the AuE surface and adopted a hairpin conformation. A complementary telomer labelled at 5′ terminus was hybridised to the hairpin conformer which in the presence of telomerase and dNTPs extend the primer, hybridising with the hairpin removing the MB label from the surface. The ratiometric sensor had a good dynamic range of 0.2–200 cells µL^−1^ with and calculated LOD of 0.02 cells µL^−1^. The correlation coefficient of 0.992 exhibited the reliability of the biosensor.

#### 2.1.2. Selectivity Strategies

DNA-based biosensors already boast impressive selectivity towards target DNA, with aptamer and antibody-based probes similarly selective. However, when single point mutations in the DNA sequence can have a profound biological effect and significant biomedical implications, improving selectivity remains a key research goal. Duplex stability improves with hybridisation stability; however, this relationship breaks down for longer DNA strands. Probing this stability often requires the use of high temperature near the duplex melting point or chemical denaturing of the strands [32], making the incorporation of either into biosensors unfeasible, and alternative strategies are required to discriminate single point mutations.

**Figure 10:**
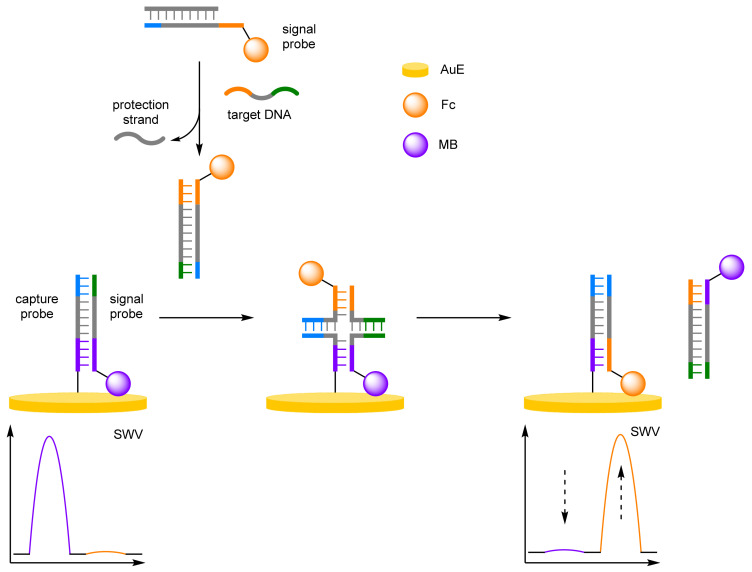
Schematic representation of a biosensor for the detection of single point mutation via DNA branch migration.

Multiple methodologies have been developed to obtain the selectivity required for single point mutation discrimination, and their incorporation with ratiometric electrochemical technique has led to reliable, accurate biosensors. Xie et al. demonstrated that cascade DNA branch migration could be employed with dual-redox labels for selective and robust DNA detection (Figure 10) [33]. A two-component approach was utilised with a 3′-MB label at the end of one signal probe, which was complementary to a capture probe immobilised onto a AuE, and a second signal probe labelled 3′-Fc, which was complementary to that of a protection strand. In the absence of target DNA, the protection strand would hybridise with the Fc signal probe, preventing it from interacting with the capture probe. This capture probe would be hybridised to the MB signal probe, placing the redox label close to the electrode. In the presence of target DNA, competitive hybridisation with the Fc signal probe occurs, revealing toeholds, overhanging single-stranded DNA complementary to another strand of single-stranded DNA. This then initiates spontaneous strand displacement, via a Holliday junction, with the hybridised MB signal-probe/capture-probe duplex, which itself has complementary toeholds. This displaces the MB signal probe into solution by the Fc-labelled strand, which in turn places the Fc label in proximity to the electrode. DNA concentration was determined using the sum of the change in current from both MB and Fc (Δ*I_Fc_* + |Δ*I_MB_*|). A correlation coefficient was determined to be 0.997 and an LOD was estimated to be 85 pM. More importantly, the system could easily distinguish between complementary DNA and mutant-type DNA, which included a base pair mismatch, a base insertion, and a base deletion.

(a) Locked Nucleic Acids

**Figure 11:**
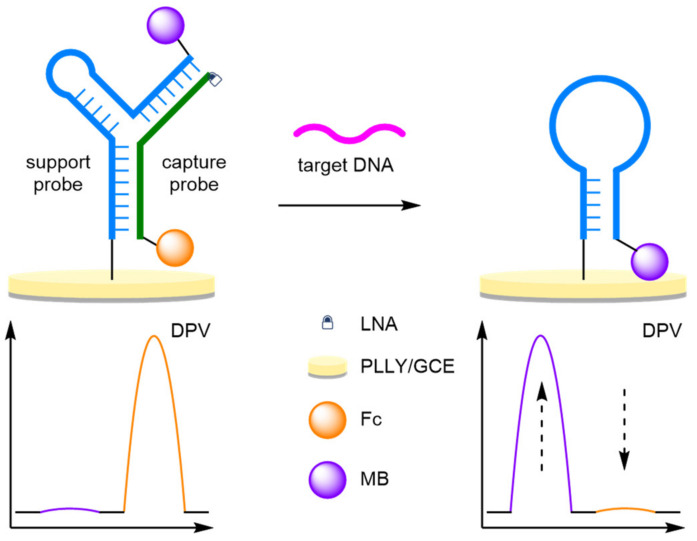
Schematic representation of a biosensor for the detection of single point mutation via locked nucleic acid strand displacement reaction.

Locked nucleic acids (LNAs), conformational restricted RNA nucleotides, improve the thermal stability of base pairings, with a resultant increase in selectivity making their incorporation into biosensors favourable [34,35,36]. An amplification-free methodology for the detection of miRNA was developed by Chen et al. using LNA-assisted strand displacement reaction (LSDR) (Figure 11) [37]. A Y-shaped molecular beacon was formed from two complementary DNA strands: a MB-labelled support strand immobilised onto a AuE and a Fc-labelled probe DNA strand. The Y conformation positions the Fc label close to the electrode, with the MB label remaining distal. Target miRNA binding to the Fc-labelled strand initiates LSDR, destroying the Y-structure, with the MB-DNA hairpin conformation reforming, resulting in an increase in MB signal. The LNAs impart increased stability and improve the rate of strand displacement, allowing for single mismatched DNA differentiation. The unamplified strategy affords an impressive LOD at 2.3 fM, with a small RSD of 2.15% over eight repeats at 50 fM concentration. The variance over 10 samples was small at 0.147, compared to the single-signal biosensor with reported variance of 0.401 and 0.262 for *I_MB_* and *I_Fc_*, respectively. This simple biosensor reported a comparable LOD to amplified methodologies, demonstrating the selectivity imparted by LNAs.

(b) Triple-Helix Molecular Beacons

Another strategy to improve the selectivity of DNA biosensors is the construction of triple-helix molecular beacons (THMBs) [38]. In general, an electrochemically labelled hairpin strand is immobilised onto the DNA surface, and subsequent addition of the capture DNA strand induces formation of the THMB. Two short complementary sequences on the capture probe bind to the hairpin strand, removing the initial label from the electrode surface. THMB possess similar stabilities to DNA duplexes. However, the increased length of free target sequence confers an increased selectivity towards the target DNA. If the capture strand is also labelled, then ratiometric electrochemical sensing is possible, combining increased selectivity with improved reliability and reproducibility.

**Figure 12:**
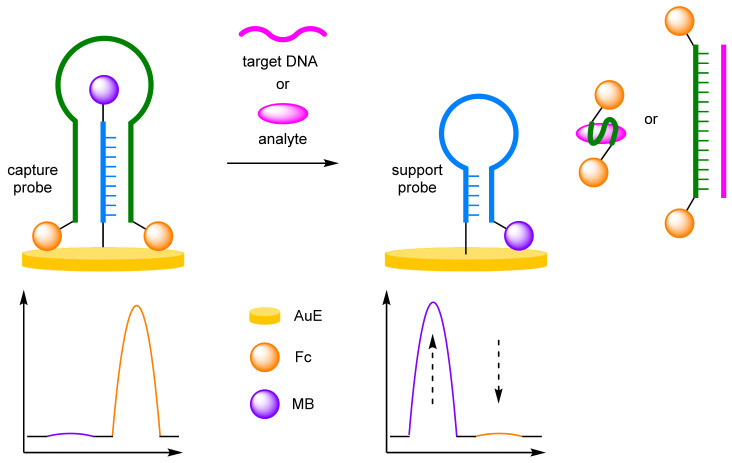
Schematic representation of triple-helix molecular beacon-based biosensor for either target DNA or analyte detection.

One of the first reported THMB-based ratiometric biosensors was by Zhang and Chen et al. who used a MB-labelled hairpin probe, and a doubly labelled Fc capture probe (Figure 12) [39]. On target DNA binding, hybridisation with the probe strand removes the Fc label, and reformation of the hairpin structure results in an increase current arising from MB. Ratiometric analysis based upon the two currents (*I_MB_*/*I_Fc_*) was possible over a relatively small dynamic range of 0.5–80 pM, with a correlation coefficient of 0.985, and a calculated LOD of 0.12 pM, in the same order of magnitude as the single-signal LODs. However, the more important selectivity of the biosensor was reported, with the ability to differentiate between single-base mismatched and triple-base mismatched DNA at a concentration of 50 pM, with a 47.9% and 25.5% signal intensity. The selectivity of THMB can be further improved with the introduction of signal amplification strategies, with a significant increase in selectivity reported by Xiang et al. with single mismatched DNA displaying similar currents to the background test [40]. Single-base-mismatch discrimination factors, the ratio between signal from the perfectly matched DNA to a single-base mismatched strand, were reported between 40 and 58, a significant improvement compared to previous electrochemical biosensors. Triple-helix molecular beacons are not only limited to DNA biosensors, with Huang et al. reporting an aptamer-based sensor for adenosine triphosphate (ATP) (Figure 12) [41]. The molecular beacon is constructed in the same fashion, with the aptamer adopting the beacon conformation around the universal MB-labelled DNA strand. The assay offered impressive reproducibility with an RSD of 2.9% over 20 experiments, with a 2.5% RSD reported from five electrodes. An LOD of 5.2 fM was calculated, in the same order of magnitude compared to the ‘switch-off’ assay, and improved the reliability of the ratiometric system, with a correlation coefficient of 0.998 compared to 0.989.

##### DNA Four-Way Junctions

**Figure 13:**
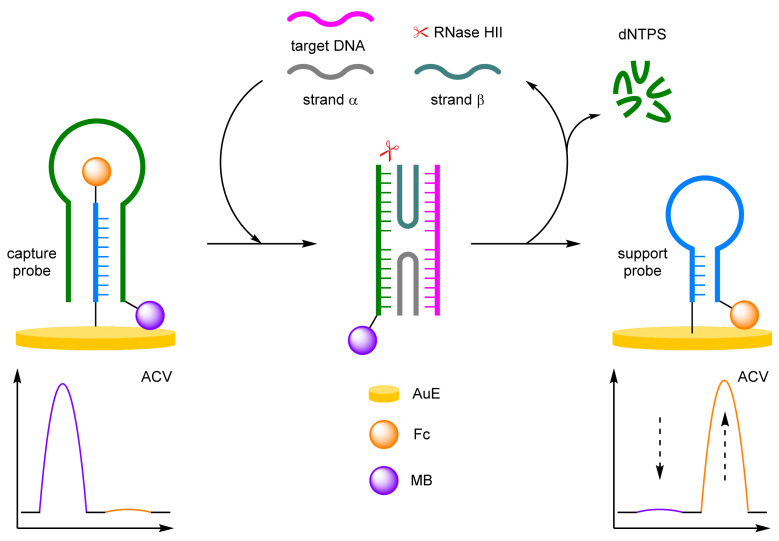
Schematic representation of a biosensor for the detection of single point mutation via DNA four-way junctions.

Another strategy to improve the selectivity of DNA sensors are DNA four-way junctions (DNA-4WJ), where their capability to discriminate single-nucleotide differences at ambient temperatures is a distinct advantage [42,43,44]. The first group to incorporate the DNA-4WJ into a ratiometric electrochemical probe was Zhang et al., who utilised an enzyme-assisted recycling amplification in a generic DNA sensor (Figure 13) [45]. A THMB was formed from a universal Fc-labelled strand immobilised onto a AuE, with a complementary MB-labelled capture probe hybridised. Two unmodified strands, α and β, which are partially complementary to the MB hairpin and the target DNA strand, remained free in solution. In a positive assay, DNA-4WJ formation occurs with the α and β strand, and the capture probe, removing the redox label from the electrode surface. The universal probe then forms a hairpin conformer bringing the Fc label close to the surface. An exonuclease digest the capture probe allowing for the formation of a new DNA-4WJ, amplifying the signal. Selectivity studies with single mismatched DNA strand exhibited low electrochemical switch-on compared to the target DNA, demonstrating the benefits of DNA-4WJs. The reproducibility of the ratiometric biosensor was investigated with an RSD of 2.1% calculated (n = 6) at a 1 nM target DNA concentration.

#### 2.1.3. Amplification Strategies

Increasing the sensitivity of an assay can be achieved through employment of a compatible amplification protocol [46,47]. More recent efforts in electrochemical assay development have been towards successfully combining amplification methods with ratiometric electrochemical detection. Traditional methods include label amplification, where the multivalent nature of nanoparticles increase the number of signal-reporting molecules upon a successful analyte-recognition event, and target amplification, where a target can generate more than one signal-producing molecule through multiple analyte-recognition events [48,49,50,51].

**Figure 14:**
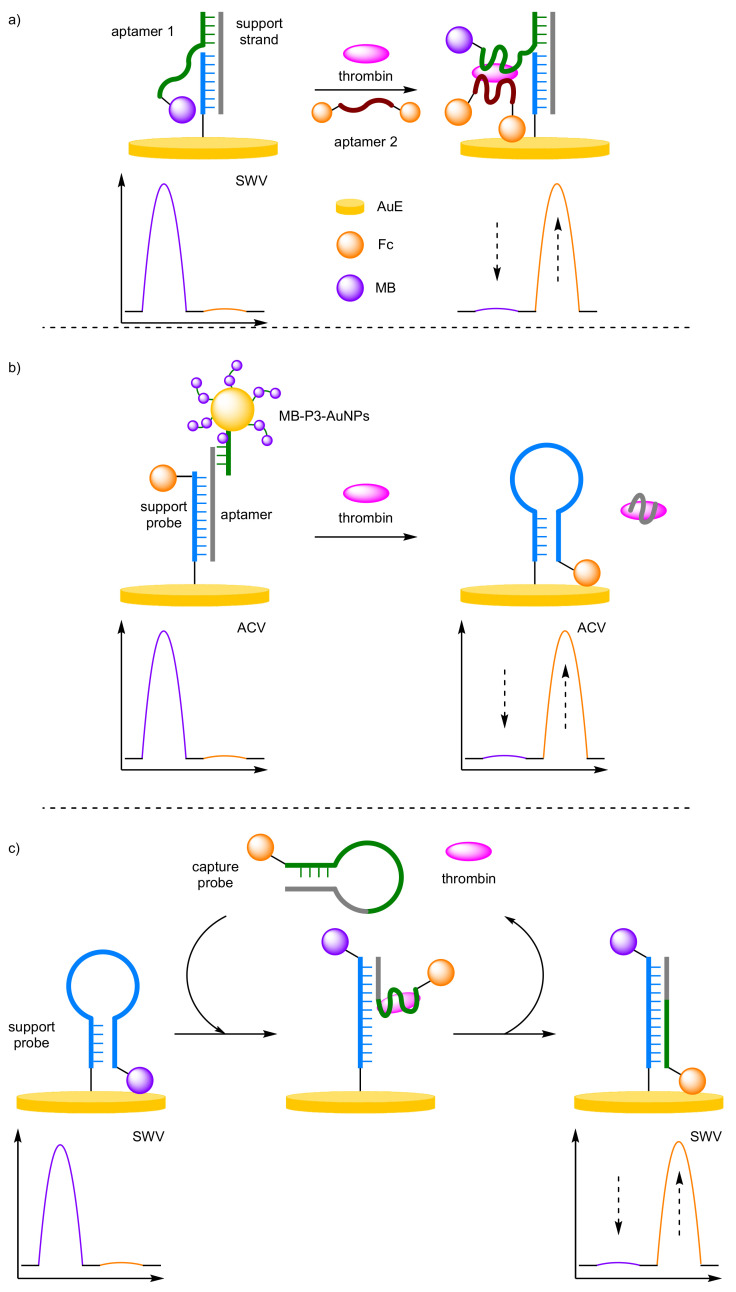
Schematic representation of three biosensors for the detection of thrombin: (**a**) non-amplified aptasensor; (**b**) label amplification-based aptasensor; (**c**) target amplification-based aptasensor.

Approaches towards the non-amplified and amplified ratiometric electrochemical detection of thrombin, a coagulation protein, can provide the perfect illustration of the benefits afforded by amplification. Firstly, Zhang and Chen et al. used a dual-aptamer sandwich structure to generate a non-amplified, ratiometric electrochemical thrombin aptasensor (Figure 14a) [52]. A double-stranded DNA was used as the solid-support on a AuE to which a thrombin-specific aptamer was appended and labelled at its overhanging 5′ end with MB. In the absence of the protein, the anchored aptamer is free to move, allowing the label to be in proximity to the electrode, detectable by SWV. In the presence of the target aptamer binding removes the redox label from the electrode causing a decrease in current. The addition of a secondary DNA aptamer, labelled at both ends with Fc, leads to further target-induced binding, placing the second redox-active labels close to the electrode and causing an increase in current. A dynamic range for thrombin concentration was determined to be 1–600 nM, with a correlation coefficient of 0.996, and a LOD of 170 pM, which is in the same order of magnitude as that of the single-redox label system. Reproducibility testing of five repeated experiments on different electrodes afforded an RSD of 3.9%.

Ma and Wang et al. were then able to show that the use of AuNPs could provide an increase in current signal response through label amplification (Figure 14b) [53]. Here, the AuNPs, multiply-labelled with MB through non-participate DNA intercalation (MB-P3-AuNPs), were bound to a AuE via a thrombin-specific aptamer and a support probe, 3′-Fc labelled. Despite their increased distance from the electrode compared with that of Fc, the label amplification provides a detectable current for MB. In the presence of thrombin, competitive binding for the aptamer occurs and the solid-supported structure is destabilised leading to removal of the AuNPs from the electrode, and a decrease in MB current. Once released, the anchor probe can undergo a conformational change to its preferred hairpin conformer, which in turn places the Fc label proximal to the electrode with an increase in current for Fc observed. The dynamic range of the system was determined to be 3 pM–30 nM, with a correlation coefficient of 0.995 and the LOD was estimated to be 1.1 pM, which is a factor of 100 more sensitive than the non-amplified method. Importantly, the ratiometric detection method delivered acceptable reproducibility, with standard deviations between 2.4% and 4.6%, over three repeated assays for three different thrombin concentrations.

Gao et al. have also demonstrated an amplified ratiometric electrochemical method for thrombin detection, but instead utilising target recycling to provide amplification (Figure 14c) [54]. In this instance, two hairpin DNA probes are employed: one as a target capture probe containing a thrombin-specific aptamer sequence and labelled with Fc, and the other as a support probe which is solid supported onto a AuE. The latter contains a sequence specific to that of the target capture probe and is labelled with MB. In the absence of the target, signal current is only observed at the E_ox_ of MB indicating that both probes remain in their preferred hairpin conformers. In the presence of the target, interaction between the capture probe and thrombin occurs, which opens the probe and makes the sequence specific to the support probe available. This then initiates the opening of the support probe, facilitating strand displacement of the capture probe from the protein to the support probe. The resulting solid-supported double-stranded duplex has the MB label distal and the Fc label proximal to the electrode, allowing for ratiometric analysis. During the strand displacement process, thrombin is recycled back into solution, allowing it to interact with more capture probes thus causing an amplification in signal. This enabled a further improved LOD of 41 fM over a dynamic range between 0.1 pM and 10 nM, and a correlation coefficient of 0.995. When considering only one of the redox labels, correlation coefficients of 0.959 and 0.910 were observed with Fc and MB, respectively, despite the LODs being very similar. This provides further evidence that the dramatic increase in accuracy and confidence when moving from a single redox label method to a ratiometric, or dual redox label method.

(a) Enzyme-Assisted Amplification

To improve the sensitivity of the dual-labelled DNA detection assay, classic DNA amplification methods have been shown to be compatible with ratiometric electrochemical endpoint detection. Enzyme-assisted DNA amplification methodologies have been a cornerstone of biosensor developments, where their high activity and facile reconfiguration to new specific analytes have been favourable. However, they are often limited by their high cost, complexity, and potential issues with contamination, which inhibits their incorporation into biomedical and point-of-use devices.

##### Exonuclease

**Figure 15:**
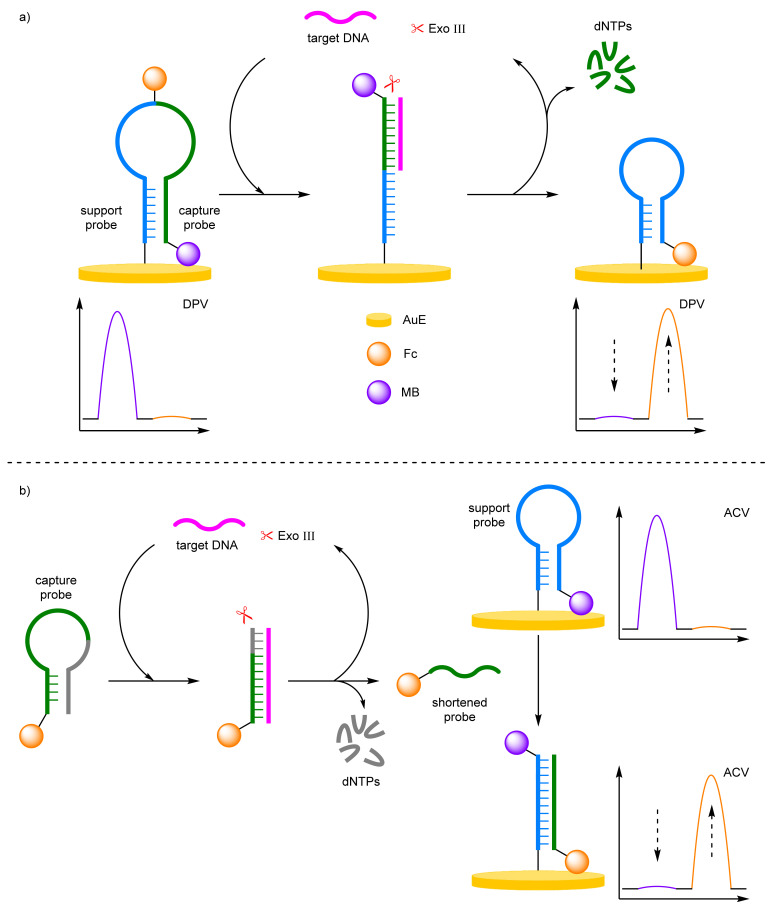
Schematic representation of two exonuclease-based target recycling amplification biosensor: (**a**) a heterogeneous amplification step; (**b**) a homogeneous amplification step.

Exonuclease III (Exo III) is a sequence-independent duplex-specific enzyme that has limited activity against single-strand DNA. The enzyme possesses a high exodeoxyribonuclease activity in the 3′ to 5′ direction and has been utilised in biosensors to selectively digest duplexes for target recycling amplification strategies. For example, Chen et al. demonstrated that an exonuclease-assisted target recycling amplification strategy can be used to amplify the signal and therefore improve the sensitivity of the biosensor (Figure 15a) [55]. In this instance, a single-stranded DNA capture probe was immobilised onto a AuE at its 5′ end, and labelled at its midpoint with Fc and at the protruding 3′ end with MB. In the absence of the target, the probe adopts a hairpin conformation, placing the MB label close to the electrode and the Fc label away from the electrode. In the presence of the target, a double-stranded duplex is formed with the capture probe. Exo III then digest the probe strand recycling the target strand back into solution. The terminal MB redox label is also removed, and the remaining probe strand forms another hairpin conformer, which places the Fc redox label close to the electrode. Target DNA is identified through the decrease in MB current and the increase in Fc current, discernible via DPV. The ratiometric detection method gives an improved LOD by an order of magnitude over using the current observed for a single redox label and offers a dynamic range between 10 and 800 fM, with a correlation coefficient of 0.985. Importantly, the ratiometric detection method again displayed good reproducibility, by affording an RSD of 4% when five individual electrode setups were exposed to 1 pM of target DNA.

By binding the DNA probe to the electrode, the enzyme kinetics are inhibited, as hydrolysis occurs at the electrode surface where configurational and spatial limitation become apparent. Ma and Wang et al. adopted a similar exonuclease-assisted target recycling amplification strategy [56]. However, by separating the two redox labels onto different strands, a homogeneous system was designed with an unbound capture probe labelled 5′-Fc in solution, and a second support probe complete with 5′-MB label immobilised on the AuE (Figure 15b). On target DNA hybridisation with the capture probe, the duplex is digested by the exonuclease, recycling the target DNA and releasing a shortened Fc-labelled probe. The shortened probe hybridises with the bound probe, opening the conformer. This moves the MB label away from the electrode surface, and moves the Fc label close, with the change in current monitored via ACV. The probe offered a significant improvement in dynamic range from 20 fM to 2 nM. This improvement is attributed to the advantageous homogenous system over the heterogeneous equivalent, with a comparable LOD reported of 12.8 fM. The biosensor exhibited good reproducibility across three repeated detections at target concentration across three orders of magnitude with a reported RSD of ~3%.

##### Strand Displacement Polymerase Reaction

**Figure 16:**
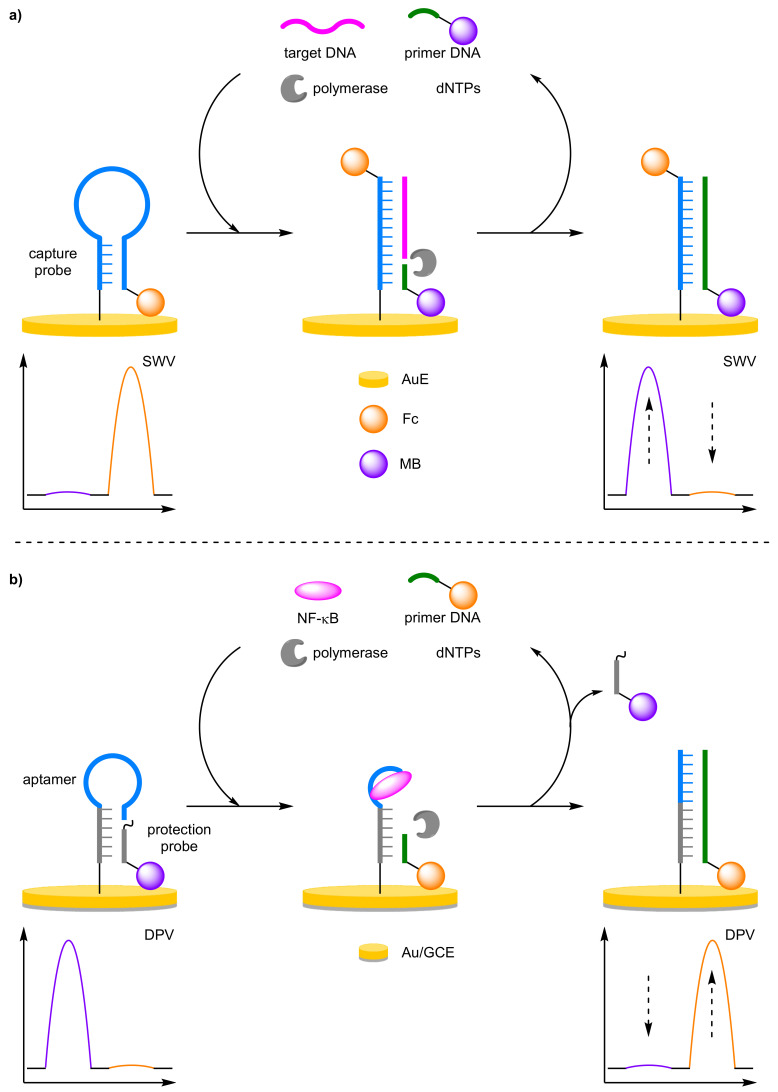
Schematic representation of two polymerase-base target amplification biosensor: (**a**) for target DNA; (**b**) an aptasensor for NF-κB.

Biosensors based upon strand displacement polymerase reaction offer significant increase is sensitivity through target amplification. Gao et al. implemented a target DNA recycling strategy with a ratiometric electrochemical detection method using a two redox-labelled component, molecular beacon-mediated circular strand displacement assay (Figure 16a) [57]. Here, the molecular beacon capture probe was labelled 5′-Fc and immobilised onto a AuE. An 8 mer polymerase primer labelled 5′-MB, complementary to the capture probe, remained in solution distal to the electrode. In the absence of the target, the probe strand adopts a hairpin conformer which places the terminal Fc redox label close to the electrode. In the presence of the target DNA, hybridisation forms a double-stranded duplex removing the Fc label and the primer can anneal. DNA polymerisation from the MB-labelled primer is then initiated through the addition of dNTPs and DNA polymerase leading to target recycling. The concentration of target DNA could be determined by the ratio between the increasing MB current and decreasing Fc current. This allowed for a greatly improved assay reliability with a correlation coefficient for the correlation equation of 0.998 determined over the course of five independent assays. This is compared to values of 0.957 and 0.958 when measuring the current of only a single redox label, either Fc or MB, respectively. Additionally, the assay displayed a large dynamic range (100 fM to10 nM) and an estimated LOD of 28 fM, two orders of magnitude lower than that of the same assay performed without amplification from polymerase-catalysed target recycling. Zhang et al. showed that an aptamer-based ratiometric electrochemical aptasensor for nuclear factor kappa B (NF-κB) could be designed along the same principles (Figure 16b) [58]. The aptasensor afforded an LOD of 30 fg mL^−1^, with a correlation coefficient of 0.987 over a dynamic range of 0.1 pg mL^−1^–15 ng mL^−1^. The reproducibility of the aptasensor was relatively good; with an RSD of 8.8% over five experiments and 9.1% over five electrodes.

##### Rolling Circle Amplification

**Figure 17:**
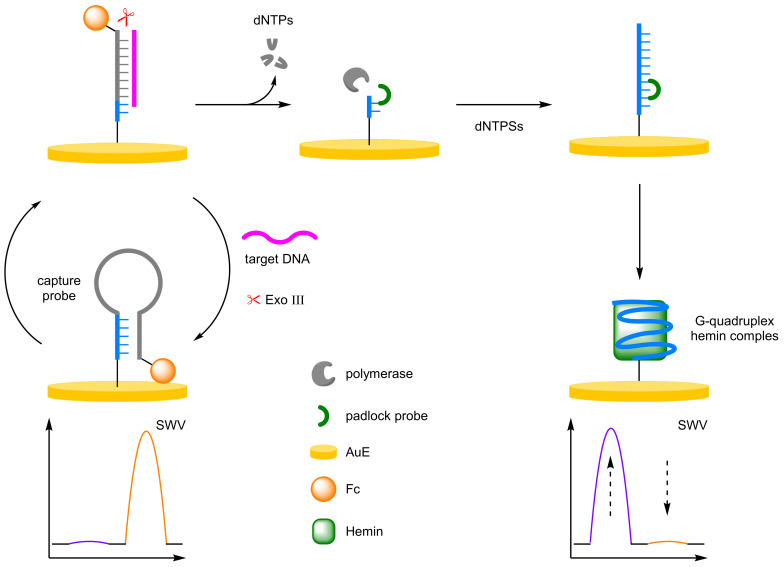
Schematic representation of a rolling circle amplification-based biosensor.

Rolling circle amplification (RCA) is an alternative amplification method used in biosensor development [59,60]. RCA requires a primer, a circular DNA template, and dNTPs in combination with a polymerase to form an elongated single-stranded DNA. The single-stranded DNA can then bind multiple labels to amplify the signal. Huang et al. combined exonuclease-assisted target recycling with rolling circle amplification in an strategy for the detection of K-ras gene (Figure 17) [61]. A 3′-Fc-labelled capture probe was immobilised onto a AuE, where it adopted a hairpin conformer bringing the Fc close to the electrode surface. Addition of the target DNA opens the hairpin conformer, and the exonuclease can then cleave the DNA duplex, removing the Fc label and recycling the target DNA. The residual single-stranded oligomer can then be elongated via RCA, where binding of the padlock probe templates the formation of an extended structure containing multiple tandem-repeat guanine base sequences. The single-stranded DNA then forms a stable G-quadruplex which subsequently binds hemin, with formation of the G-quadruplex-hemin complex monitored via DPV at −360 mV (*I_hemin_*). Determination of target DNA concentration was calculated from the ratio of the current between the decreasing Fc and increasing hemin. The assay had a dynamic range of 0.5 fM–10 pM, with a LOD of 0.28 fM. This is only slightly lower than when using either *I_Fc_* or *I_hemin_* individually, with a LOD of 0.7 and 0.6 fM reported, respectively. The reproducibility of the assay was highlighted by the authors with an RSD of 1.9% reported after 15 successive scans, and the RSD across five electrodes was 2.1% at 10 pM.

##### Catalytic Cascades

**Figure 18:**
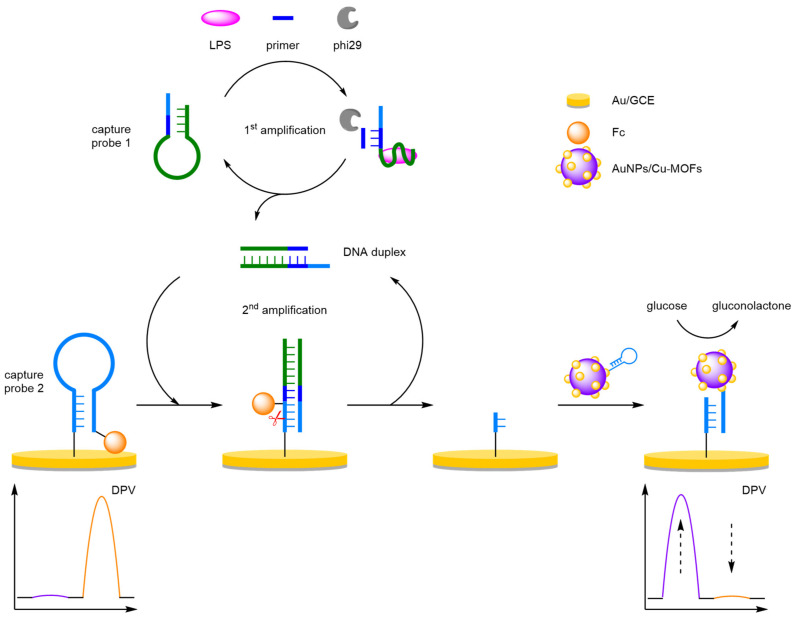
Schematic representation of a catalytic cascade amplified biosensor.

For maximum sensitivity within a diagnostic assay, catalytic cascades are essential to provide significant signal amplification for a detectable signal at ultralow analyte concentrations. Zhuo and Yuan et al. provide an excellent demonstration of such a catalytic cascade for signal detection (Figure 18) [62]. The catalytic cascade involves a target-initiated, target-recycling protocol which triggers a subsequent catalytic cycle to reveal binding sites for a signal-amplifying copper catalyst with a distinct electrochemical signal. The target, a lipopolysaccharide (LPS), causes a conformational change to the capture probe DNA strand to reveal a DNA primer sequence. From this primer, polymerase-catalysed elongation generates double-stranded DNA, which then acts as a signal transducer, and recycles the analyte. The DNA duplex is then able to hybridise with an immobilised hairpin capture DNA labelled 3′-Fc. The resulting DNA superstructure reveals a specific cleavage site which is selectively trimmed using a nicking endonuclease, releasing the Fc redox label into solution, and recycling the DNA duplex. Once trimmed, the remaining solid-supported DNA strands act as binding sites for AuNPs-labelled Cu-MOFs. Upon the addition of a solution of glucose, signal current is observed using DPV at an E_ox_ of −180 mV, generated by Cu-MOF-catalysed glucose oxidation. In the absence of the target, the catalytic cascade is not initiated, and the capture DNA probes are not nicked, leaving the Fc redox labels near the electrode with an E_ox_ observed at 160 mV (*I_Fc_*). The signal amplification catalyst cascade enabled a LOD of 0.33 fg mL^−1^ and using the ratio between the two electrochemical signals, a linear dynamic range between 1 fg mL^−1^ and 100 ng mL^−1^, with a correlation coefficient of 0.996.

(b) Toehold Mediated Strand Displacement Reaction

**Figure 19:**
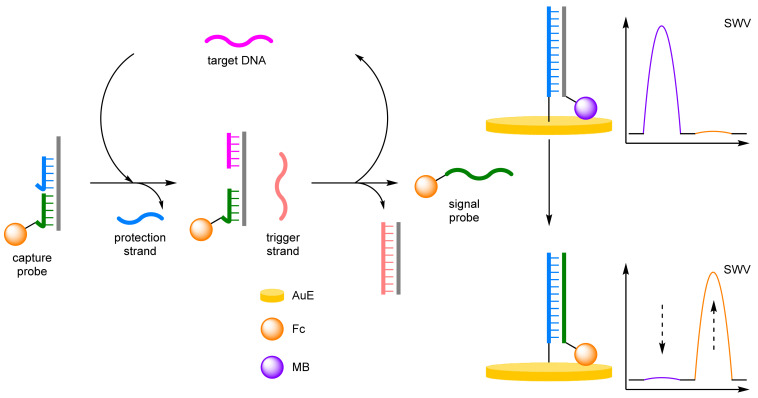
Schematic representation of toehold mediated strand displacement reaction.

Toehold mediated strand displacement reaction (TMSDR) utilises an overhanging section of DNA, formed when a duplex contains one chain shorter than the other, and the increased stability of duplexes containing more complementary base pairs [63]. This combination leads to spontaneous strand displacement, initiated in the toehold region to the thermodynamically more stable duplex. Xie et al. developed a TMSDR-based biosensor for the detection of target DNA (Figure 19) [64]. Firstly, a capture probe was formed from a support strand hybridised with both a protection strand and the Fc-labelled probe. A MB-labelled duplex was immobilised onto a AuE, and a trigger strand was left unbound. In the presence of target DNA, TMSDR occurs between the target DNA and the capture probe, releasing the protection strand and exposing a second toehold section. This exposure allows for the trigger strand to hybridise, triggering a second TMSDR, which releases the signal probe and recycles the target DNA. The released signal probe undergoes a final TMSDR with the immobilised duplex, releasing the MB-labelled strand from the electrode surface and bringing the Fc label into proximity. The TMSDR cascade afforded an improved LOD compared to previous methods, with a high correlation coefficient of 0.994. A discrimination factor of 7.2 was reported for single base pair mismatched DNA. Combined with a low RSD of 1.59%, this demonstrated the excellent reproducibility and selectivity of the biosensor.

(c) Catalytic Hairpin Assembly

**Figure 20:**
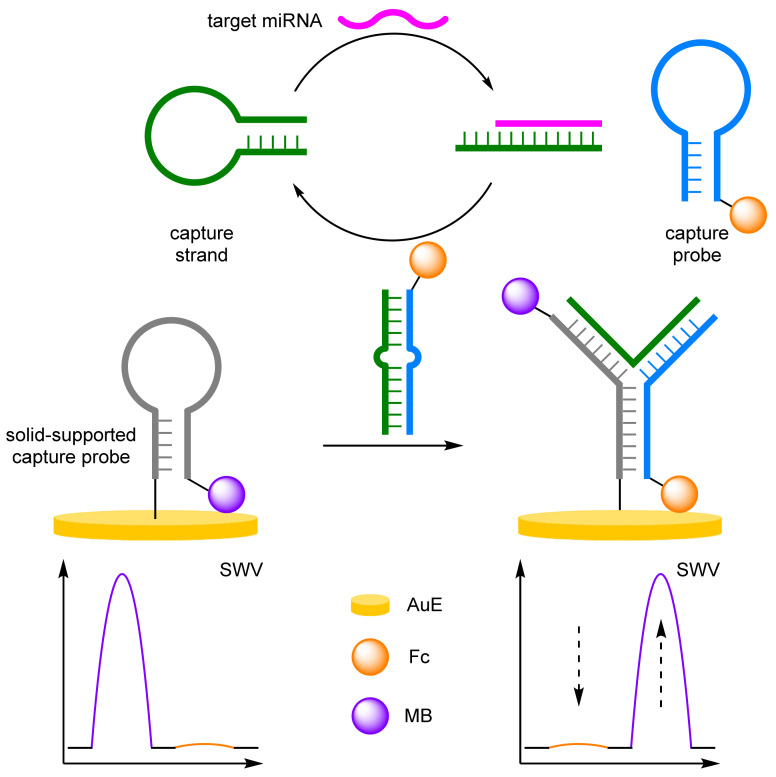
Schematic representation of catalytic hairpin assembly-based biosensor.

Catalytic hairpin assembly (CHA) offers an alternative approach to miRNA sensing. CHA features enzyme-free amplification, overcoming the associated stability issues, shortening the number of assay steps required, and simplifying the sensing environment, desirable for inclusion into point-of-use devices. The introduction of mismatched nucleotides by Ellington has improved the signal to noise ratio, previously a limiting factor for CHA, improving the selectivity of the biosensors [65,66]. Work by Xiang et al. utilised a AuE with an immobilised 3′-MB-labelled DNA capture probe (Figure 20) [67]. The second probe, labelled 3′-Fc, remained distal to the electrode in the absence of target miRNA, with a final unlabelled capture strand adopting a hairpin conformation in solution. In the presence of miRNA, hybridisation occurs with the capture strand, opening the conformation and exposing a toehold section. TMSDR by the Fc-labelled probe affords a duplex and recycles the miRNA. The duplex is then able to hybridise with the solid-supported capture probe forming a Y-shaped triplex, with the conformation removing the MB label from the electrode and positioning of the Fc label close to the surface. The reliability of the biosensor was demonstrated by its high correlation coefficient of 0.988, and the LOD of 1.1 fM was comparable with enzyme-assisted amplification strategies.

(d) DNA Walkers

**Figure 21:**
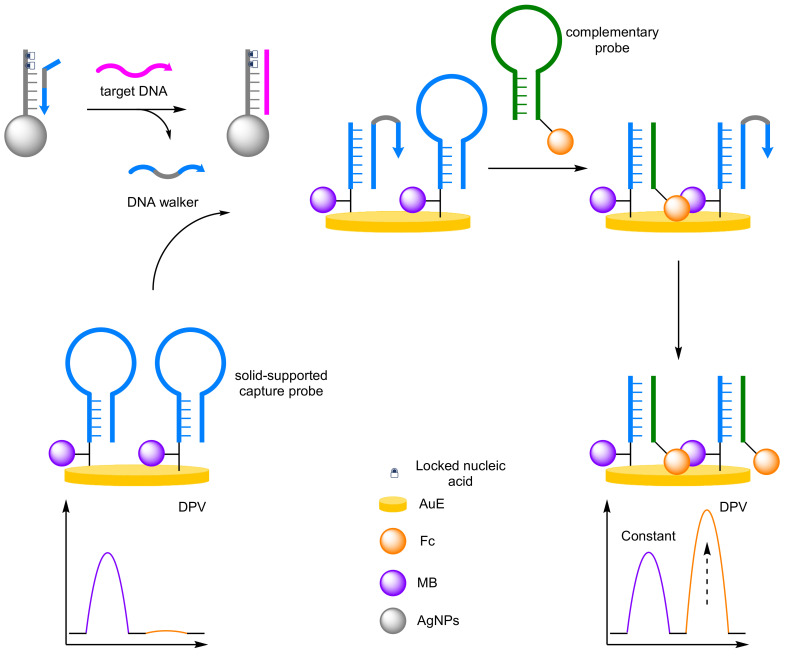
Schematic representation of a DNA walker-based biosensor.

Significant signal amplification can be achieved using DNA walkers, nanomachines that are able to ‘walk’ down DNA tracks [68,69]. The nanomachines contain two strands bound together that can open successive molecular beacons through hybridisation. TMSDR with a secondary DNA probe releases one strand of the walker that can then hybridise another molecular beacon allowing the walker to ‘walk’ down the DNA track [70]. Chen et al. developed a DNA walker system for the detection of miRNA (Figure 21) [71]. The assay combined a AuE immobilised with proximally MB-labelled DNA probes that adopted a hairpin conformation, with a magnetic bead complete with capture probes that were hybridised with the DNA walker. In the presence of the target miRNA, locked nucleic acid strand displacement of the DNA walker occurs, and the unbound walker then ‘walks’ down the DNA track. The opened hairpin then hybridises with a complementary Fc-labelled DNA probe via TMSDR, and the DNA walker is then able to open another hairpin. The signal at −200 mV (*I_MB_*) remained constant, with an increase at 360 mV (*I_Fc_*) observed via DPV. The reproducibility of the assay was explored, with an inter-assay RSD of 4.47% and an intra-assay RSD of 3.21% from five repeats. The advantages of the ratiometric system was demonstrated with a high correlation coefficient of 0.993, a four order of magnitude dynamic range from 0.1 fM to 100 fM, and an attomolar LOD of 67 aM. Xie et al. extended DNA walker methodology beyond DNA utilising aptamers for small molecule detection [72]. A similar approach was taken by Chen and Zhang et al. However, they incorporated an exonuclease to improve the sensitivity of the assay for thrombin [73]. Further progression has seen the incorporation of three-dimensional DNA walker which imparts greater mobility, overcoming the conventional shortcomings of two-dimensional walkers and resulting in improved sensitivity of the biosensors [74].

(e) Metal Nanoparticles

**Figure 22:**
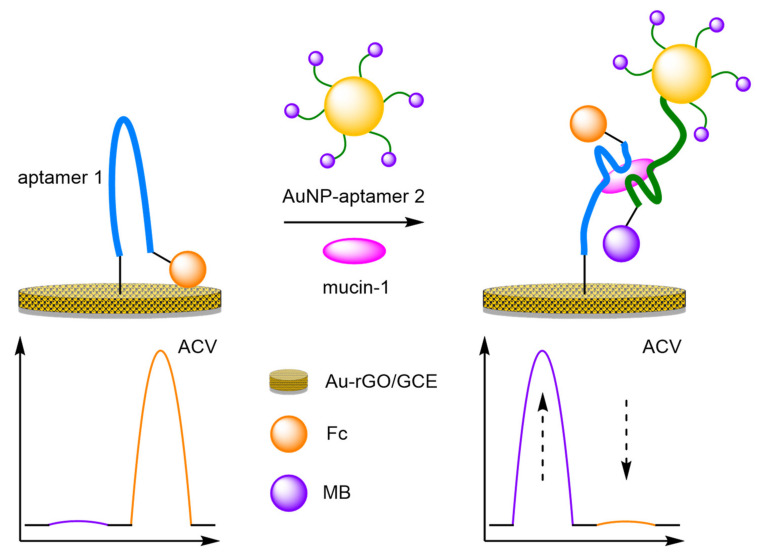
Schematic representation of a nanoparticles-assisted label amplification biosensor.

The introduction of gold nanoparticles (AuNPs) into biosensors has become a popular choice, especially in the design of new modified electrodes, but their incorporation into DNA-based biosensors remains less common. Their inclusion offers two key advantages. Firstly, the addition of AuNPs to the electrode structure improves the electrical conductivity of the electrode, improving sensitivity. Secondly, the AuNPs create anchor points for DNA attachment for non-gold electrode surfaces. AuNPs have already demonstrated their utility as amplification agents for the detection of thrombin. A further example of the amplification properties of AuNPs can be seen in the sandwich-type immunoassay utilised by Wang et al. to improve the sensitivity for the detection of mucin-1 (Figure 22) [75]. An AuNP-reduced graphene oxide (rGO) composite was electrodeposited onto a GCE, creating a large surface area. A 5′-thiolated aptamer was immobilised onto the electrode, with a 3′-Fc label, and separately, AuNPs were labelled with 3′-MB-labelled aptamers. In the absence of mucin-1, the Fc remains close to the electrode surface with the MB signal minimal. However, on analyte-aptamer binding, the Fc label is removed from the surface with a decrease in signal observed. Binding of the AuNP-aptamer in a sandwich-type assay brings the MB labels closer to the electrode. Repeatability studies showed an RSD of 5.7% (n = 6) at 1 nM, and the assay displayed an improved sensitivity compared to non-amplified biosensors, producing a calculated LOD of 0.25 pM, with a large dynamic range of 1 pM–1 µM.

(f) Multiple Label Intercalation

**Figure 23:**
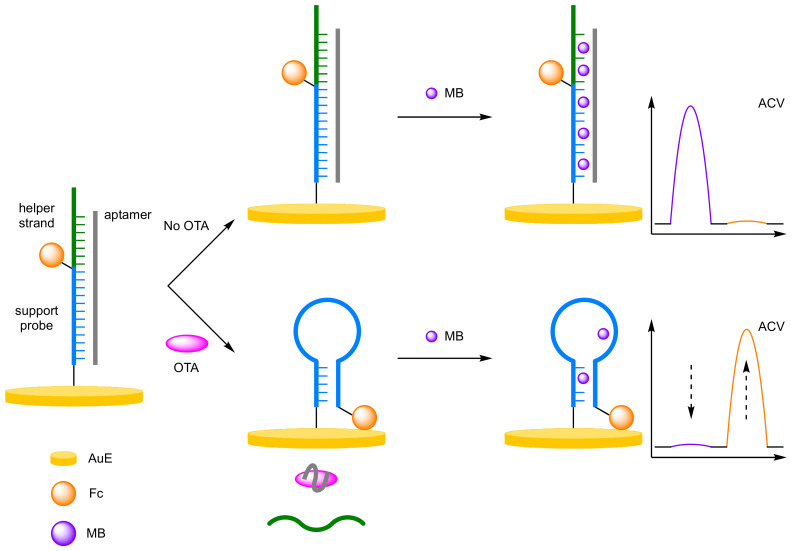
Schematic representation of multiple label intercalation-mediated amplification aptasensor for the detection of Ochratoxin A (OTA).

The difference in intercalating properties of label molecules into single-stranded DNA compared to double-stranded DNA can be used to incorporate multiple label molecules into duplexes in a label amplification biosensor [76]. MB is more readily intercalated into double-stranded DNA than single-stranded DNA, and You et al. used this property for the detection of Ochratoxin A (OTA) (Figure 23) [77]. A 3′-Fc-labelled DNA support probe, complementary to the aptamer for OTA, was immobilised onto a AuE. Hybridisation with the unbound aptamer and a helper strand of DNA elongated the double-stranded DNA. Intercalation of MB into the duplex afforded a reference peak. On analyte addition, aptamer binding occurs, destroying the superstructure and the support probe adopts a hairpin conformation. As MB intercalates better into double-stranded DNA, the MB signal decreases on aptamer binding, allowing for ratiometric analysis between the Fc and MB signals. The reliability of the assay was demonstrated with a correlation coefficient of 0.995 reported. The ratiometric assay offered an increased dynamic range (10 pg mL^−1^ to 10 ng mL^−1^), and an LOD of 3.3 pg mL^−1^, in the same order of magnitude compared to the single-signal equivalent. The reproducibility of the assay was demonstrated with an RSD of 1.9% over six electrodes.

(g) Hybridisation Chain Reaction

**Figure 24:**
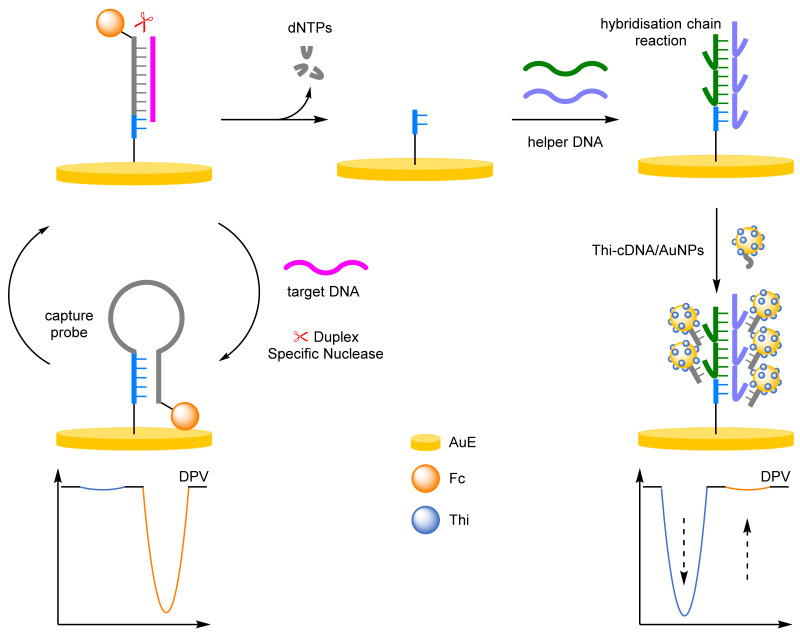
Schematic representation of a hybridisation chain reaction-based biosensor with nanoparticle-assisted label amplification.

The hybridisation chain reaction (HCR) is an enzyme-free strategy for improving sensitivity through label amplification [78,79,80]. HCR is the hybridisation of two strands of DNA that is triggered in the presence of a target DNA strand, polymerising in an alternating end-over-end fashion. The extended duplex can then intercalate multiple labels, giving significant label amplification. Liang and Qiu et al. reported a dual-amplification strategy for the detection of miRNA; combining a duplex-specific nuclease and an HCR to obtain unparalleled amplification (Figure 24) [81]. The Fc-labelled capture probe was attached to the AuE and adopted a hairpin conformation with a peak at 470 mV (*I_Fc_*) observable by DPV. On target hybridisation the hairpin opens, removing the Fc label. A duplex-specific nuclease cleaves the DNA duplex leading to the recycling of the miRNA. The residual DNA acts as a primer to initiate the HCR, forming an extended duplex from polymerisation of the two helper DNA strands. The duplex contains multiple overhanging sections that an AuNP-bound capture DNA strand hybridises with. The AuNPs contain multiple thionine (Thi) redox labels that, on hybridisation, are brought close to the electrode surface, increasing in the signal at −230 mV (*I_Thi_*). The dual-amplification strategy afforded impressive sensitivity with a LOD of 11 aM and a dynamic range of 100 aM–100 pM. The reproducibility of the biosensor was explored at 10 pM concentrations, with an RSD of 2.7% reported across six batches. HCR can also be used for the detection of enzymes, for example Miao et al. was developed for the detection of human 8-oxoguannine DNA glycolase 1, which utilised HCR in combination with a ruthenium redox label [82]. The ratiometric assay offered an improved dynamic range than the single-signal system with a range of 0.002–10 U mL^−1^, compared to 0.01–10 U mL^−1^.

**Figure 25:**
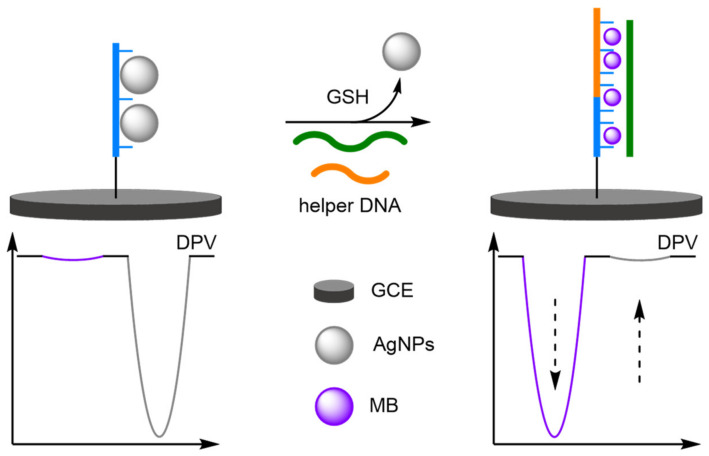
Schematic representation of a hybridisation chain reaction-based biosensor with intercalation-assisted label amplification.

DNA-templated metallisation was discussed previously as a popular technique for the formation of well-defined metal nanoparticles, but the resulting structures can destroy the recognition properties of the DNA [83]. This can be restored through the addition of thiols, a mechanism which has been utilised to full effect by Qu et al. for the ratiometric electrochemical detection of glutathione (GSH), a thiol-containing small molecule important to biological processes (Figure 25) [84]. Here, single-stranded capture DNA probe strands were first attached to a modified GCE, which served as a template for the formation of silver nanoparticles (AgNPs). In the absence of a thiol target, the AgNPs prevent the initialisation of a hybridisation chain reaction (HCR) and a sole Ag/Ag^+^ oxidation peak is observed at 150 mV. In the presence of GSH, the high binding affinity of the thiol for silver results in the removal of the AgNPs from the probe DNA strand, and HCR is initiated. The DNA superstructure is then electrochemically detected using MB as a DNA intercalator. An increase in current at the E_ox_ of MB with a concomitant decrease in current for the AgNPs is observed under a positive assay scenario. The assay displayed a linear dynamic range between 10 and 1000 nM, with a correlation coefficient of 0.997 and a LOD of 0.10 nM.

#### 2.1.4. Summary

It is clear to see that DNA is by far the most utilised and versatile structure when developing ratiometric electrochemical biosensors. This is not surprising, due to the ease in which custom sequences with specific redox-active labels can be acquired. The emergence of DNA aptamers as analyte-recognition molecules has extended their application to not just DNA, but also to other important analytes of interest. These range from small molecules, such as drugs, to heavy metals, like mercury. In the majority of articles discussed, the deployment of ratiometric detection methods that use two redox-active species has clearly provided an increase in reliability and reproducibility, in comparison to their singly labelled counterparts. However, in some assays, more rigorous testing is needed to demonstrate the robustness of the biosensor prior to its potential adoption within an applied setting. Ratiometric electrochemical detection methods have also shown to be compatible with numerous different amplification strategies, which enables the development of highly sensitive biosensors whilst ensuring reliable electrochemical endpoint detection.

### 2.2. Modified Electrodes

A popular way of introducing an electrochemical internal reference label, as opposed to secondary labelling of DNA architectures, is to directly modify or label the electrode itself. Typically, this is achieved through one of three different ways: direct labelling of the electrode with a redox-active material or tag that has a distinct electrochemical signal; modification of the electrode with macromolecular analyte-recognition structures for host/guest-enabled displacement assays using two different redox-active labels; and modification of the electrode surface with oxidation catalysts which serve to produce distinct electrochemical signals in the presence of specific small molecules.

#### 2.2.1. Direct Labelling of Electrodes

**Figure 26:**
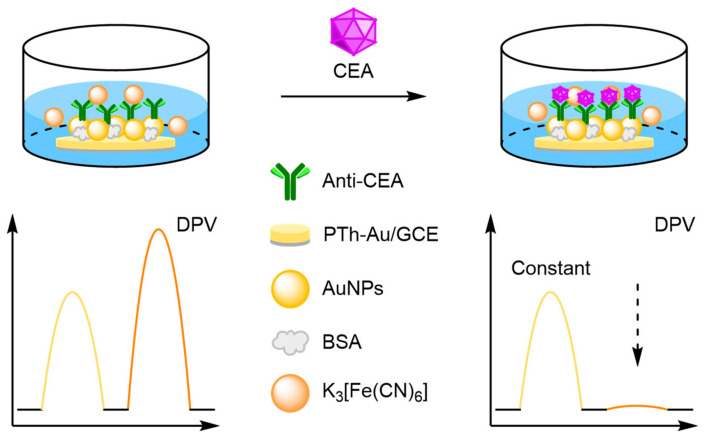
Schematic representation of a modified electrode for the detection of carcinoembryonic antigen (CEA).

One approach towards modifying the electrode with an internal reference is to use an electrochemically active material as the electrode surface. Weng and Lin et al. were one of the first to demonstrate this technique by employing a polythionine- and gold-modified electrode material to develop an electrochemical immunoassay for tumour biomarker detection (Figure 26) [85]. To achieve this, a GCE was first modified with a pre-prepared polythionine-gold (PThi-Au) composite prior to the subsequent addition of AuNPs and an antibody specific for carcinoembryonic antigen (CEA). Before the addition of the analyte, the electrodes were tested with potassium ferricyanide solution using DPV and two peaks were observed: one for the polythionine-modified electrode, and one for ferricyanide. Upon addition of the target, the ferricyanide peak decreases due to both steric and electrostatic effects preventing the ion from reaching the electrode. In contrast, the current observed for the polythionine remains identical, showing its excellent potential as an electrochemical internal reference electrode material. The ratio between the two peaks could then be used to accurately determine analyte concentration. The ratiometric electrochemical method was then extensively tested with 30 experiments conducted on 10 electrodes over multiple days. This resulted in a vastly improved average standard deviation and variance of 0.044 and 0.002, respectively, in comparison to the non-ratiometric method, which afforded 2.81 and 7.86, respectively. The ratiometric method also exhibited a higher correlation coefficient of 0.997, in comparison to 0.995, over a linear range between 5 and 40 pg mL^−1^ of CEA and a LOD of 1.7 pg mL^−1^. However, the current response of the internal reference electrode material was found to be less desirable, especially in comparison to the ferricyanide ion, and its E_ox_ was also found to shift in different pH buffers, potentially limiting any future application.

**Figure 27:**
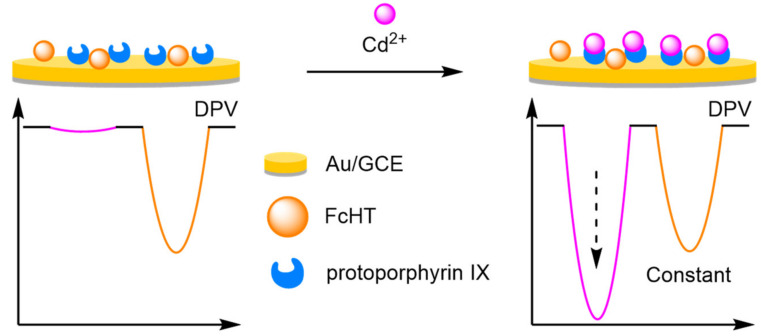
Schematic representation of a modified electrode for the detection of Cd^2+^.

To improve the signal of the internal reference, Tian et al. utilised the facile electrochemical properties of Fc for the ratiometric electrochemical detection of heavy metals (Figure 27). An AuNP-modified GCE was labelled with both ferrocene hexanethiol (FcHT) as the internal reference and a ligand with a high binding affinity for the analyte of interest. Protoporphyrin IX was chosen as the ligand, due to its high binding affinity for cadmium [86]. In the presence of increasing concentrations of the target, DPVs displayed steadily increasing cadmium peaks while the peak corresponding to Fc remained identical, demonstrating that a ratiometric electrochemical method had been successfully developed. A linear working range was found between 0.1 and 10 μM, and an LOD of 10 nM achieved. Reproducibility experiments were conducted over six different electrodes and delivered an RSD of 3.2%. The approach was found to be general, as the porphyrin ligand could be changed to a pyridine-containing tetramine ligand. This enabled the ratiometric electrochemical detection of copper, which found application for the in vivo monitoring of copper in rat brain [87].

(a) Nanoparticles

**Figure 28:**
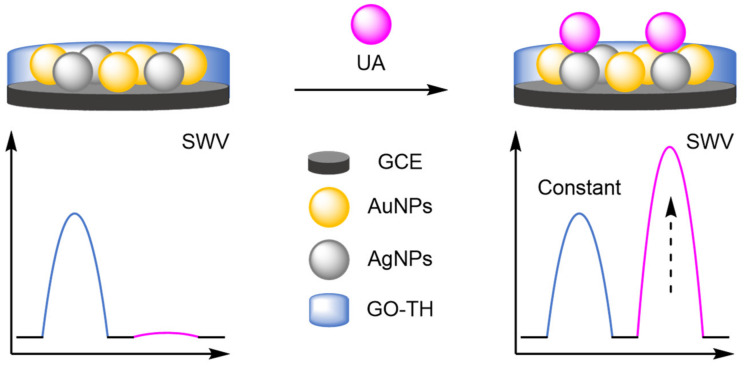
Schematic representation of a metal nanoparticles-based modified electrode for the detection of uric acid.

Nanoparticles provide anchor points for DNA immobilisation and function as amplifiers for the electrochemical label in DNA-based biosensors, and they have served the same purpose in the development of new modified electrodes. The catalytic activity of analytes can be improved by the inclusion of metallic nanoparticles, allowing for the direct detection of electroactive molecules [88,89,90]. When combined with an internal reference, ratiometric electrochemical analysis is possible, which significantly improves the reliability of the biosensors. The prevalent use of nanoparticles has become a recurring theme in the development of new electrodes. For example, a bimetallic approach was adopted by Gui and Wang, who functionalised a GCE with AuNPs and silver nanoparticles (AgNPs) (Figure 28) [91]. These nanoparticles enhanced the oxidation of uric acid (UA) at the electrode surface, with an increase in current at 460 mV observed by SWV. Electrostatic adsorption of a graphene oxide-thionine composite (GO-Thi) via π–π stacking provided an internal reference at −280 mV (*I_Thi_*). This allowed reliable electrochemical analysis, with a correlation coefficient of 0.993 over a dynamic range of 1–100 µM. The LOD calculated was comparable to previously reported single-signal sensors. However, the reproducibility was demonstrated with an RSD of 2.6% obtained from six separate electrodes. A similar approach adopted by Wang et al. involved using the specific analyte binding affinity of creatine for copper nanoparticles (CuNPs) in their sensor [92]. The ratiometric method exhibited two dynamic ranges, 0.01–0 µM and 10–100 µM, with a calculated LOD of 2 nM, and good reproducibility of 1.9% RSD. Structurally similar biosensors have been developed for phenol with molybdenum or manganese [93,94], for neurotransmitters with manganese [95], and organic dyes with titanium [96].

**Figure 29:**
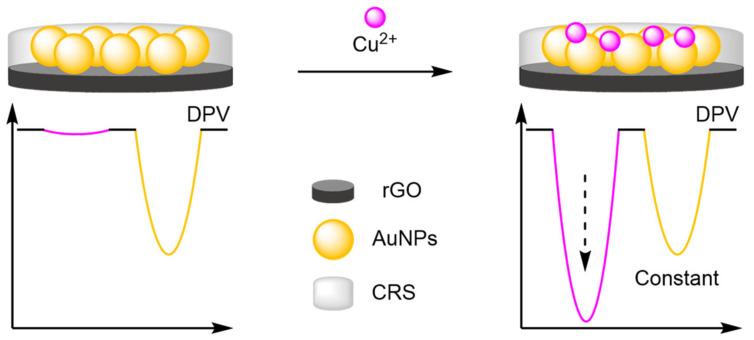
Schematic representation of a resin-supported nanoparticle-based modified electrode for the detection of Cu^2+^.

The stability of nanoparticle-modified electrodes is an important consideration when designing them; where aggregation and detachment of nanoparticles greatly hinders the stability of the electrodes, preventing their facile incorporation into point-of-use devices. Therefore, several strategies have been explored to improve their long-term stability, repeatability, and reproducibility. Luo and Yang embedded AuNP into a carbonised resin as an internal reference for the detection of Cu^2+^ (Figure 29) [97]. 15 measurements were conducted over five days, with 92% of the original signal maintained. Furthermore, across 10 repeat experiments, an RSD of 3.8% was recorded and inter-assay RSD of 3.4% across five electrodes. By comparison, the RSD without the internal reference was 13.2%, displaying the improved reliability of ratiometric sensing. A superhydrophobic electrode approach was the cornerstone of the approach by Li et al., where a zeolite-imidazole framework was formed creating a superhydrophobic surface [98]. The increased hydrophobicity prevented non-specific absorption, an important factor in complex sample testing and in electrode longevity. The long-term stability was demonstrated, with >94% maintenance of the original signal after 30 days, along with good intra- and inter-assay reproducibility (RSD of 3.5% and 1.7–3.1%, respectively) for the detection of multiple analytes including adrenaline, serotonin and tryptophan.

(b) Metal Organic Frameworks

**Figure 30:**
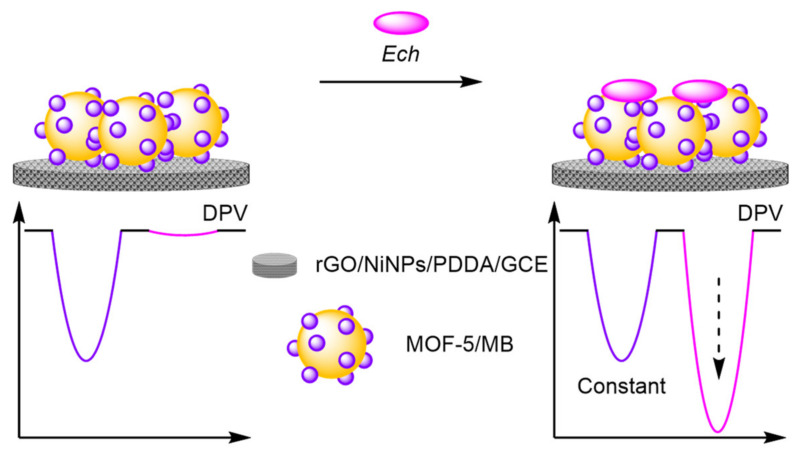
Schematic representation of a MOF-based modified electrode for the detection of echinacoside.

An alternative method developed for encapsulating reference molecules was to functionalise the electrode surface with metal-organic frameworks (MOFs) and utilise their porous morphology to encapsulate multiple redox labels. Ye et al. formed a nanocomposite consisting of nickel nanoparticles (NiNPs), graphene oxide (GO), and poly (diallyldimethylammonium chloride) (PDDA), with MOF-5 self-assembled in situ to form the nanocomplex that was then immobilised onto a GCE (Figure 30) [99]. MB was encapsulated into the structure to act as an internal reference with a constant signal at 30 mV. The assay displayed excellent reliability, with a correlation coefficient of 0.991 for the ratiometric detection of echinacoside (*Ech*) compared with 0.977 from the single ‘switch-on’ assay. Reproducibility studies demonstrated good stability with an inter-assay RSD of 3.52% over five electrodes and intra-assay across six repeats of 4.46%. The LOD reported was similar to the ‘switch-on’ assay, but with an increased dynamic range and reliability demonstrating the benefits of ratiometric assays.

(c) Graphene Oxide

**Figure 31:**
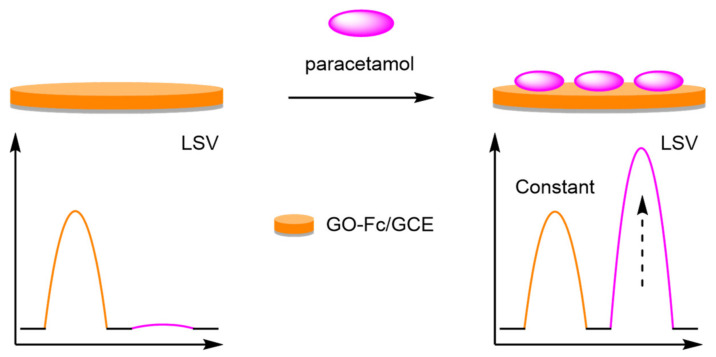
Schematic representation of graphene oxide-based modified electrode for the detection of paracetamol.

The introduction of graphene oxide (GO) onto the glassy carbon electrode (GCE) has allowed for the incorporation of an internal reference through electrostatic interactions. Guo et al. drop-coated a Fc-GO-Nafion complex onto a GCE for the detection of paracetamol (Figure 31) [100]. The addition of Nafion improved the stability of the complex, which formed the reference signal with an E_ox_ at 230 mV. Paracetamol (PA) had a distinct oxidation peak at 560 mV which was observed via linear sweep voltammetry (LSV), and the two peaks produced were then used to calculate the analyte concentration. The electrodes afforded a good dynamic range between 1 and 100 µM and an LOD of 0.2 µM. The simplicity of fabrication was also paramount in work by Gu et al., who constructed a GO-GCE labelled electrostatically with MB [101]. The resultant electrode was utilised in the measurement of cerebral ascorbic acid in in brain microdialysate, displaying excellent selectivity compared to other electroactive chemicals present in cerebral fluid. An RSD of <1% was recorded for six electrodes for the online repetitive determination of ascorbic acid concentration, displaying the high reproducibility of the assay.

(d) Carbon Nanotubes

**Figure 32:**
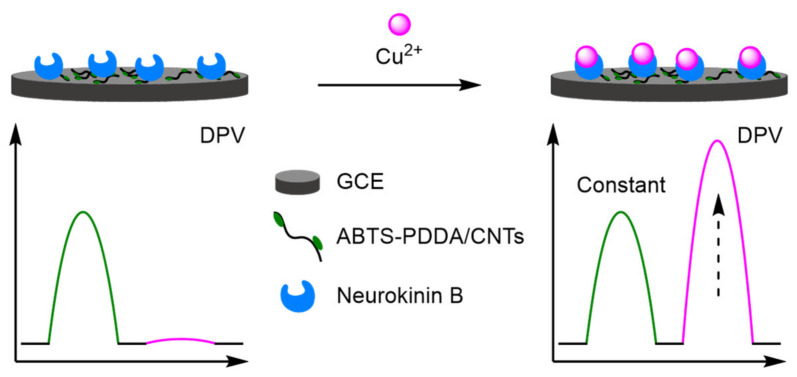
Schematic representation of a carbon nanotube-based modified electrode for the detection of Cu^2+^.

Functionalised carbon nanotubes (CNTs) are a prevalent strategy facilitating the construction of new electrodes. CNTs enhance electron transfer, improve the surface area, and can encapsulate an internal reference [102,103]. Prior to functionalisation, CNTs are limited by the number of binding sites preventing sufficient incorporation of support materials, which can reduce their catalytic activity and stability. However, CNT-containing nanocomposites can overcome these limitations, though often a decrease in conductivity is observed. Yin et al., building upon their previous work in copper sensing with poly(ionic liquid) [104], constructed a nanocomposite material from CNTs, PDDA, and 2,2′-azinobis-(3-ethylbenzothiazoline-6-sulphonate) (ABTS) which was electrodeposited onto a GCE to provide a constant reference peak (Figure 32) [105]. Embedding of a Cu^2+^ recognition element, neurokinin B, into the composite allowed for the ratiometric detection of Cu^2+^. The peak at 580 mV (*I_ABTS_*) remained constant, with a Cu^2+^ peak at −120 mV increasing over a dynamic range of 0.1–10 µM. This produced an excellent correlation of 0.991, and an LOD of 0.04 µM. The electrode was further utilised in the detection of β-amyloid peptides, which bind to the copper ion reducing the peak intensity at −120 mV in the DPV. Experiments into the electrode reproducibility demonstrated low RSD for Cu^2+^ at 4.9%, increasing to 7.1% for β-amyloid peptides. Wang et al. harnessed the electroactivity of multi-walled carbon nanotubes as an internal reference at 170 mV, for the accurate detection of dopamine (DA) at 400 mV [106]. This led to a calculated LOD of 0.23 µM, with a correlation coefficient of 0.998 over a relatively small dynamic range of 1–20 µM. However, the reproducibility of the electrode was demonstrated with an intra-electrode RSD of 0.7%, and an inter-electrode RSD of 5.0%, over five electrodes. Other modified CNTs include Fc labelling for the detection of nitrophenols [107], and phytohormones [108], and nitrogen-doped nanosheets for the detection of metal ions [109].

**Figure 33:**
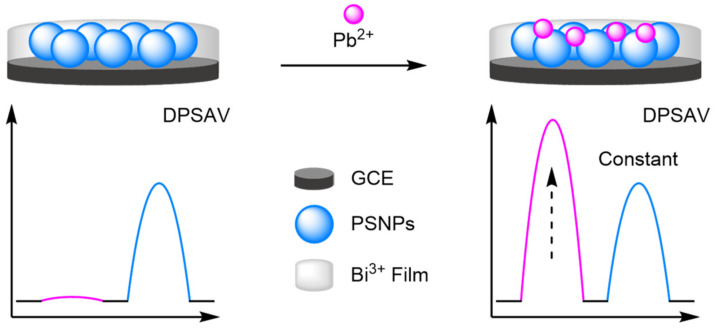
Schematic representation of Bi(III)-assisted modified electrode for the detection of Pb^2+^.

Carbon nanotubes were also integral to the detection of heavy metal ions by Shen et al. [110]. They utilised differential pulse stripping anodic voltammetry (DPSAV) for the multiplex detection of four heavy metal ions: Cd^2+^, Hg^2+^, Pb^2+^, and Zn^2+^. A conductive film was synthesised from poly(2-amino terephthalic acid) doped with CNTs and mercaptosuccinic acid. A bismuth(III) film formed in situ provided an internal reference for the sensor, with the Bi^3+^ ions forming multicomponent alloys with the metal ions. The sensor exhibited a dynamic range of 0.5–50 µg L^−1^ and showed impressive multiplex reliability, reporting correlation coefficients of 0.998 for Pb^2+^ and 0.999 for the remaining three metals. The calculated LODs ranged from 0.089 µg L^−1^ for Zn^2+^ and 0.49 µg L^−1^ for Hg^2+^. Importantly, the sensor could be readily converted for the detection of cancer biomarkers, where metal sulfide nanoparticles were utilised as distinguishable signal tags in sandwich-type assays. Following recognition, dissolving the metal sulfides released the metal ions which were then detected with a high reliability. Reproducibility studies showed that from five repeats RSDs ranged from 4.7% to 5.8% for the cancer biomarkers. Yu et al. built upon the same principles, instead using porous silica nanoparticles to support the Bi(III) coating (Figure 33). The ratiometric detection of Pb^2+^ was improved, with an LOD of 0.09 µg L^−1^ across a larger dynamic range of 0.2–100 µg L^−1^. The sensor was thoroughly tested with 40 experiments conducted on eight electrodes over five days affording an RSD of 3.6%, compared to 9.1% for the comparative single ‘switch on’ signal.

(e) Biocompatible Electrodes

**Figure 34:**
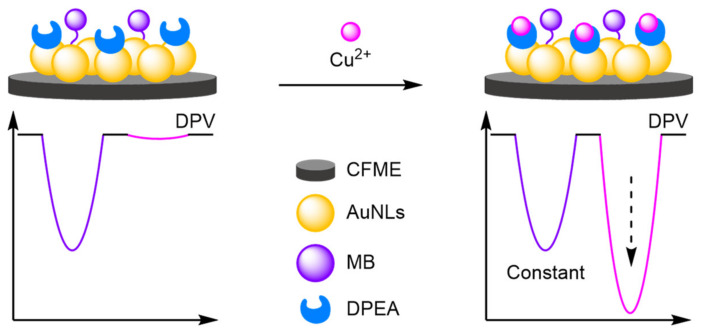
Schematic representation of a carbon fibre microelectrode for in vivo detection of Cu^2+^ and cysteine.

In vivo sensing necessitates the use of biocompatible materials for the construction of electrodes without which toxicology and stability are compromised, inhibiting the development of biomedical point-of-use devices. Carbon fibre microelectrodes (CFME) have become a prevalent strategy, where the electrodes are stable in vivo and possess minimal toxicity. Tian et al. have pioneered the field, with their research on CFME for modified electrodes and in dual-channel systems. Electrodeposition of gold nanoleaves (AuNLs) onto a CFME created a large surface area nanostructure onto which a refence MB-labelled DNA strand was immobilised (Figure 34) [111]. Cu^2+^ recognition elements were also bound with a distinct E_ox_ at 195 mV, from the MB peak at −290 mV. The dynamic range of the electrode was relatively small at 1–12 µM, with a calculated LOD of 480 nM. The inclusion of Au nanoleaves was highlighted by the authors with a 4.5-fold selectivity improvement. The electrode exploited the specific binding affinity of cysteine for Cu^2+^ to develop a compatible ‘switch off’ assay for thiols. Tian et al. further reported a CFME spun from multiwalled carbon nanotubes (MWCNTs) for the detection of oxygen and pH in brain ischemia [112]. A hemin-Fc biosensor was attached via π–π stacking to the CNF, where the Fc signal remained constant independent of oxygen concentration and pH, with the signal intensity of hemin increasing at higher oxygen concentration. In addition, the E_ox_ shifts to a more positive potential at reduced pH, allowing for the dual sensing of both variables ratiometrically. The sensor was tested in vivo displaying excellent selectivities against other neurological compounds. Further work looked at the introduction of polyethylene glycol to prevent electrode fouling, and its utilisation for the detection of furin activity in the cell [113].

#### 2.2.2. Selectivity Strategies

Selectivity in modified electrode-based assays remains a detriment to their incorporation into point-of-use applications, with a challenging transition from a clean to an uncontrolled environment. With most electrodes utilising the electroactivity of the analyte, false positives, cross contamination, and electrode fouling remain challenges to overcome in complex environments. Compared to DNA-based biosensors, where a change in the DNA code can create a novel selective biosensor, modified electrodes require different strategies to tailor the assay for a single target. Many strategies take their inspiration from DNA biosensors, incorporating aptamers and antibodies utilising their inherent selectivity to improve the assays. Alternative routes look at introducing recognition sites to distinguish between analytes to improve selectivity.

##### DNA

**Figure 35:**
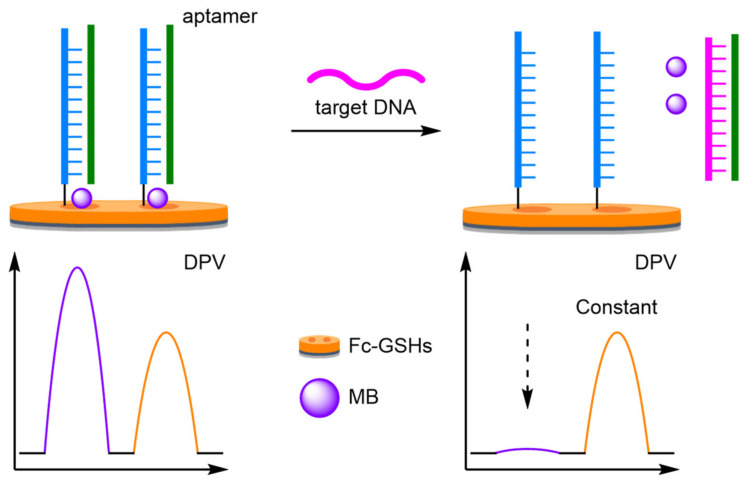
Schematic representation of a graphene-based modified electrode.

A ‘switch-off’ assay was developed by Qu et al. for the detection of target DNA, removing the requirement to label the DNA with an electroactive label, reducing the cost of the proposed biosensor and allowing for facile regeneration [114]. In this approach, graphene was modified with mesoporous silica nanomaterials before being mounted onto GCEs. This allowed molecules to be encapsulated within the silica while facilitating electron transfer (Figure 35). Ferrocene carboxylic acid was covalently bound to the sandwich structure, serving as the internal reference, before the nanomaterials were loaded with MB and sealed with duplex DNA probes. Once constructed, strand displacement of the duplex DNA probes would only occur in the presence of complementary target DNA, which would unblock the mesoporous silica channels and release MB into solution. The presence of target DNA would therefore be attributed to a decrease in current for MB, while the current for Fc remained the same. A linear dynamic range of the assay spanned six orders of magnitude between 10 nM and 10 fM, with an LOD of 10 fM. More importantly, the ratiometric method greatly improved the accuracy and reproducibility of the assay, with a correlation coefficient of 0.989 compared with a correlation coefficient of 0.981 when just measuring MB peak current. Li et al. constructed a biosensor upon similar principles, using [Ru(NH_3_)_6_]^3+^ loaded positively charged mesoporous silica nanoparticles sealed with single-stranded capture DNA, and [Fe(CN)_6_]^3−^ in solution acting as a reference signal [115]. An alternative approach combined the high selectivity of DNA and the diffusivity of single strand DNA for the detection of target DNA. Li et al. modified an indium titanium oxide (ITO) with naphthalene sulfonate, creating an electrode that has a high selectivity for single-stranded DNA over double-stranded DNA, through strong π–π stacking interactions between the nucleotide bases and the planar naphthalene [116].

##### Antibodies

**Figure 36:**
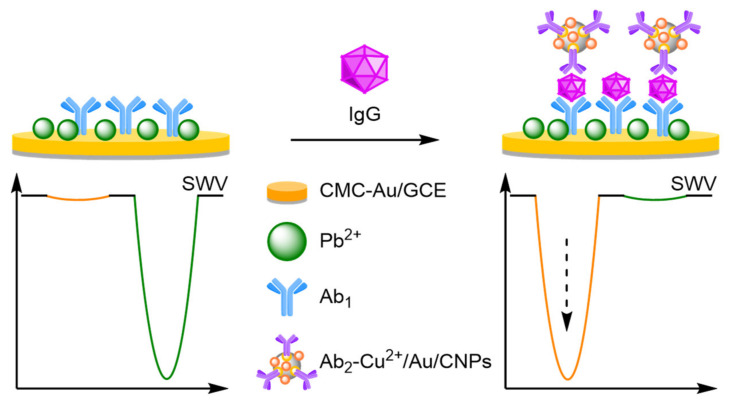
Schematic representation of an antibody-based modified electrode for the detection of immunoglobulin G (IgG).

Metal NP sandwich-style sensors can improve selectivity combining the selectivity of antibodies, with the transducing properties of NPs. Tang and Ma constructed a sandwich assay for the detection of immunoglobulin G (IgG) using two functionalised AuNPs (Figure 36) [117]. Firstly, a GCE was functionalised with a carboxymethyl cellulose-Au-Pb^2+^ nanocomposite, then labelled with the recognition antibody 1 (Ab1). Secondly, carbon nanoparticle (CNPs) were functionalised with AuNPs labelled with the other recognition antibody (Ab2). Cu^2+^ ions were incorporated into the CNPs as a reporter signal, with a Pb^2+^ oxidation peak used as a reference. In the presence of the analyte, antibody binding brings the Cu^2+^-labelled nanoparticles closer to the electrode, increasing their signal intensity, with the reference peak reducing. The assay was reliable from 1 fg mL^−1^ to 100 ng mL^−1^, with a correlation coefficient of 0.994, and a calculated LOD of 0.26 fg mL^−1^. The selectivity of the assay against common interference molecules demonstrated that even in large excess of the interferents, reliable ratiometric analysis was possible. Other similar metallic NP-based sandwich assays included a biosensor for carcinoembryonic antigen detection [118], and Li et al. showed that metal-labelled synthetic melanin nanospheres (SMNPs) could be used in the construction of biosensor for nuclear matrix protein 22 [119].

##### Aptamer

**Figure 37:**
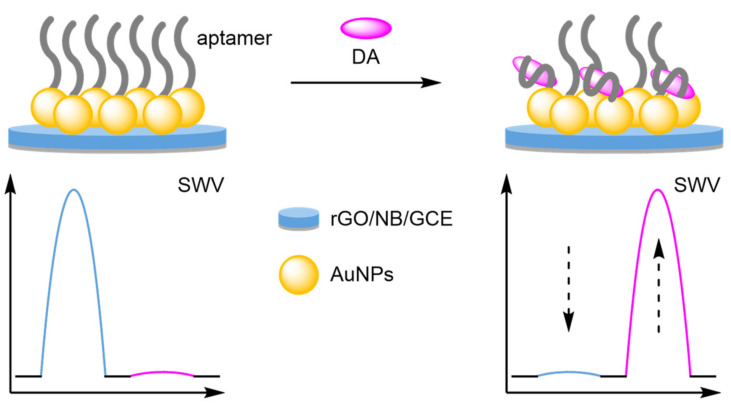
Schematic representation of an aptamer-based modified electrode for the detection of dopamine (DA).

Aptamer binding offers another strategy to expand and improve the selectivity of sensors toward novel targets. Combining a modified electrode containing an internal reference with a bound aptamer, new sensors have been developed for electroactive analytes. Gui and Wang et al. used a Nile blue (NB) internal standard with a dopamine (DA)-specific aptamer, producing a highly selective biosensor (Figure 37) [120]. Aptamer binding brings the electroactive analyte close to the electrode surface, whilst inhibiting the diffusion of other molecules to the surface. The biosensor displayed excellent reproducibility. After 15 days, six experiments afforded an RSD of 3.5%, with a correlation coefficient of 0.992 across a dynamic range of 10 nM–0.2 mM. The selectivity towards DA was explored against other neurotransmitters, which at 10-fold excesses still had minimal current changes compared to DA, confirming the selectivity expected for aptamer binding. A similar strategy was used by Deng et al. who developed an aptamer-based biosensor for 17β-estradiol using a Thi internal reference [121]. The reusability of the assay was thoroughly explored with an RSD of 10.3% recorded after 30 experiments.

##### Recognition Sites

**Figure 38:**
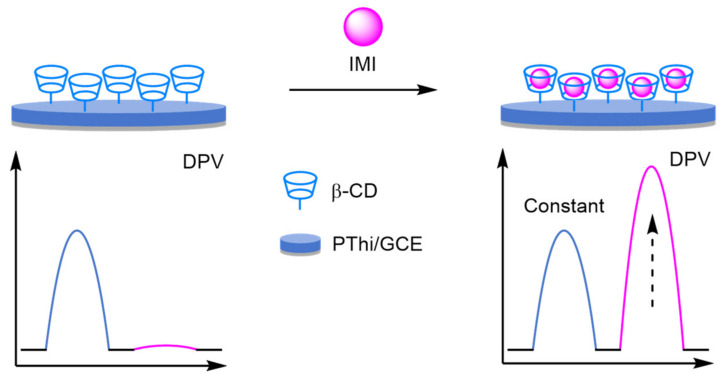
Schematic representation of β-cyclodextrin-based modified electrode for the detection of imidacloprid (IMI).

Addition of non-DNA recognition sites to electrodes have greatly reduced the complexity and cost of modified electrodes, whilst maintaining the selectivity desired for complex sampling. The incorporation of β-cyclodextrin (β-CD), which contains a hydrophobic internal cavity that undergoes host–guest cavity interaction, has allowed for specific analyte detection. This strategy was utilised by Li and Kan, who electropolymerised β-CD and Thi onto a GCE (Figure 38) [122]. Thi provided a constant internal reference at −250 mV (*I_Thi_*), with host–guest interaction of the imidacloprid (IMI), an insecticide analyte, with the β-CD affording a peak at −950 mV, allowing for ratiometric analysis via DPV. The size-selective interaction was integral to the selectivity displayed against other insecticides and the assay demonstrated excellent reliability across four orders of magnitude, with a correlation coefficient of 0.999 and a calculated LOD of 17 nM.

##### Molecular Imprinted Polymers

**Figure 39:**
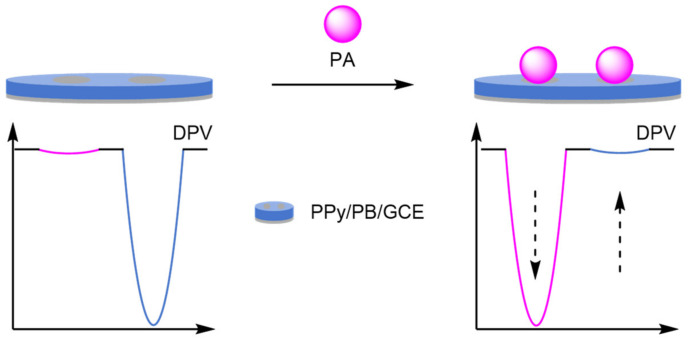
Schematic representation of a molecular imprinted polymer-based modified electrode for the detection of Paracetamol (PA).

Molecular imprinted polymers (MIPs) restrict non-specific binding, offering a cost-effective alternative to other strategies to improve assay sensitivity. The templated polymerisation forms analyte-specific sites, significantly improving the selectivity and stability of the electrode. Kan et al. developed a ratiometric electrochemical sensor for PA by forming a MIP from poly(pyrrole) (PPy) templated with PA onto a Prussian Blue (PB)-modified GCE (Figure 39) [123]. PB served as an internal reference at 180 mV, with PA binding affording an E_ox_ peak at 420 mV. Reproducibility studies showed an RSD 1.2% over ten experiments. A good dynamic range of 1 nM–0.1 mM was observed, and an LOD of 0.53 nM was calculated. Alternative MIP-based biosensors have been formed from poly(thionine), for propyl gallate and DA [124]. The poly(thionine) served a dual purpose by forming specific binding sites and acting as an internal reference.

#### 2.2.3. Host–Guest Displacement Assays

**Figure 40:**
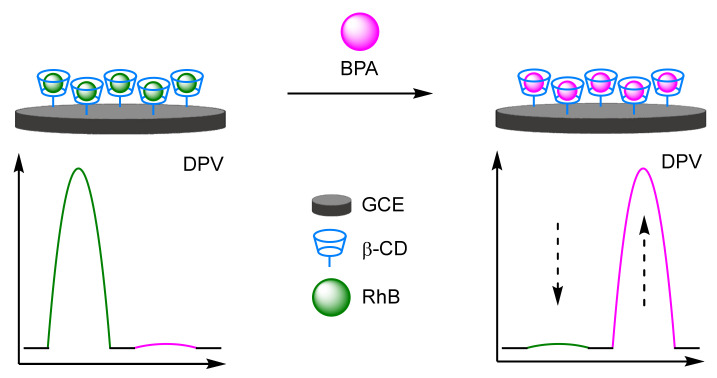
Schematic representation of a host–guest displacement assay for the detection of bisphenol A (BPA).

Host–guest displacement assays utilise the difference binding strengths of analytes towards specific host recognition sites such as β-cyclodextrin (β-CD). By loading the hydrophobic inner cavity of β-CD with the electrochemical label rhodamine B (RhB), which could be displaced by an electroactive analyte, Zhang and Chen et al. were able to utilise the setup for the ratiometric electrochemical detection of BPA (Figure 40) [125]. In the absence of the target, an E_ox_ peak at ≈900 mV (*I_RhB_*) was initially seen. In the presence of the target, this peak decreased in intensity as the RhB was displaced with BPA, thus producing an increase in current at the 575 mV (*I_BPA_*). The assay displayed a linear dynamic range between 1 nM and 6 μM, with a LOD of 52 pM. Additionally, the ratiometric method proved reproducible as a standard deviation of only 5.2% was calculated when five individually prepared electrodes were exposed to a 50 nM concentration of BPA. The same group then extended the method to electrochemically inactive proteins by using DNA aptamers tagged with MB [126]. In the presence of prion protein, the labelled aptamers bind to the target which seals the MB label inside the β-CD cavity. In the absence of the target, the aptamer can be displaced by ferrocene carboxylic acid leading to a decrease in signal for MB, and an increase in signal for the ferrocene compound. This method produced a narrow linear range of 0.2–2 pM, a correlation coefficient of 0.995 and an LOD of 0.16 pM. At a prion concentration of 1 pM, a standard deviation of 1.4% was calculated when the experiment was conducted using five individually prepared electrodes. Similar strategies have been developed for mycotoxins [127], and the detection of artificial dyes [128].

**Figure 41:**
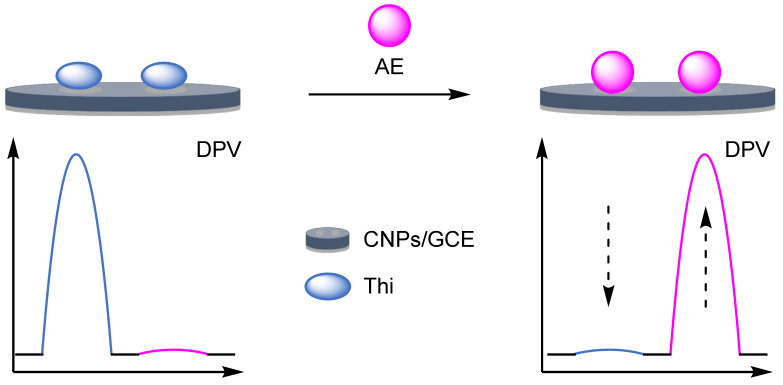
Schematic representation of a molecular imprinted polymer-based host–guest displacement assay for the detection of aloe-emodin (AE).

Kan et al. used MIPs for the detection of aloe-emodin using a host–guest displacement assay strategy (Figure 41) [129]. The electrode was formed by electropolymerisation of pyrrole templated by aloe-emodin onto CNPs. After removal of the template models, Thi could weakly bind into the cavities providing a reference signal at −130 mV. On addition of aloe-emodin, displacement of Thi occurs, causing a decrease in the reference peak. At the same time, a new analyte peak at −480 mV appears, with the change in current followed by DPV. The biosensor displayed good sensitivity with an LOD of 75 nM, with an intra-electrode RSD of 4.29% and inter-electrode at 3.35%. The selectivity expected with MIPs was confirmed, with minimal interference observed from other redox-active molecules.

#### 2.2.4. Oxidation Catalysts

To improve both the sensitivity and selectivity of ratiometric electrochemical assays, electrodes have been modified with catalysts capable of providing an amplified electrochemical signal selectively in the presence of a specific substrate. Examples of both biological catalysts, and synthetic catalysts have been described to achieve biosensors with favourable characteristics. The inherent amplification of catalysts greatly improves the selectivity and sensitivity. When combined with reliable and reproducible nature of ratiometric sensing, the afforded biosensors are excellent candidates for point-of-use devices.

##### Enzymes

**Figure 42:**
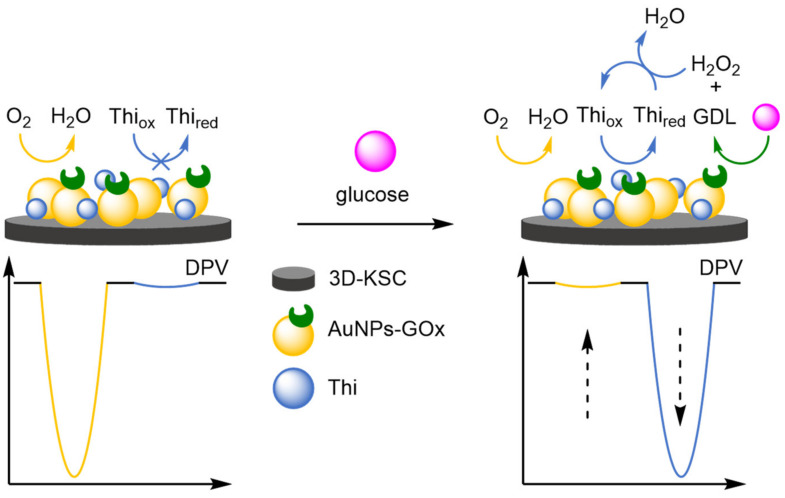
Schematic representation of a glucose oxidase-based biosensor for the detection of glucose.

Wang et al. demonstrated that glucose oxidase (GOx) could be co-immobilised on to a porous carbon electrode with AuNPs and Thi for the ratiometric electrochemical detection of glucose (Figure 42) [130]. In the absence of the target, a peak at −450 mV vs. saturated calomel electrode (SCE) was observed, which was corresponded to AuNP-catalysed reduction of O_2_. However, in the presence of glucose, a peak at −250 mV vs. SCE was observed and proposed to correspond to the Thi-catalysed reduction of H_2_O_2_, produced as a by-product of GOx-catalysed oxidation of glucose. The dynamic range obtained for the modified electrode covered three orders of magnitude from 35 µM to 15 mM, with a calculated LOD of 11.7 µM. A total of 15 biosensors were tested to determine their reproducibility with an RSD of 3.54%. In a further development of the strategy, incorporation of Cu-MOFs onto macroporous carbon followed by electrodeposition of AuNP created a functionalised electrode [131]. The AuNPs served a dual purpose, firstly catalysing O_2_ reduction as an internal reference at −500 mV, and secondly immobilising GOx. On addition of glucose, GOx catalyses its oxidation removing O_2_ from the system, thus reducing the reduction peak at −500 mV. The CuMOFs simultaneously catalyse the oxidation of glucose to glucuronic acid, with peak formation observed via DPV at −100 mV. The biosensor was comparable to their original sensor with an LOD of 14.8 µM, and an RSD across 15 electrodes of 4.52%.

**Figure 43:**
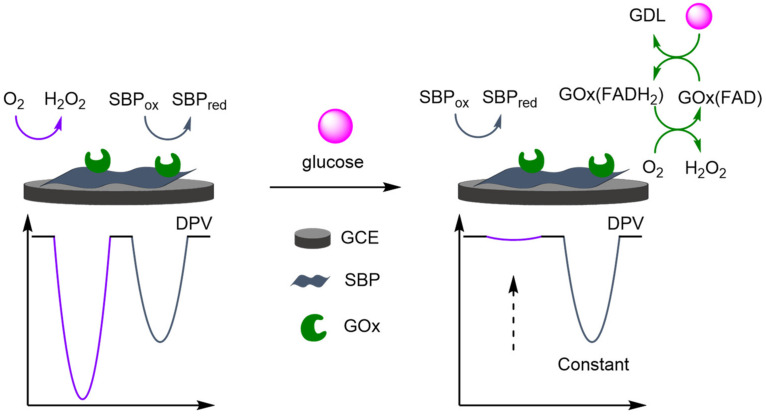
Schematic representation of a Schiff base polymer-supported biosensor for the detection of glucose.

Song et al. have utilised alternative biocompatible material in the pursuit of a glucose biosensor (Figure 43) [132]. Polymerisation of Thi and terephthalaldehyde created an electroactive Schiff base polymer (SBP) that had two separate redox peaks at −50 mV and −200 mV. Co-immobilisation of the SBP and GOx onto a GCE created the biosensor, with O_2_ reduction at the electrode with a peak at −375 mV utilised as the second signal for ratiometric analysis. On addition of glucose, the O_2_ peak decreases as the O_2_ is used to re-oxidise GOx instead of being reduced at the electrode surface. The biosensor displayed a comparable dynamic range from 0.82 µM to 4.0 mM and an improved LOD of 0.27 µM. The electrode displayed excellent reproducibility with an RSD of 0.51% across 10 electrodes, and an intra-assay RSD of 0.34% (n = 6). The flexibility of SBP was integral to its development as a wearable biosensor, which displayed good retention of LOD at 2.4 µM and a dynamic range of 7.2 µM–4 mM. The group further developed a covalent organic framework using an ammonia-aldehyde condensation reaction to form a flexible surface with two pore sizes [133]. Immobilisation of GOx and microperoxidase 11 into the dual pores allowed for ratiometric electrochemical detection of glucose.

##### Ketjen Black

**Figure 44:**
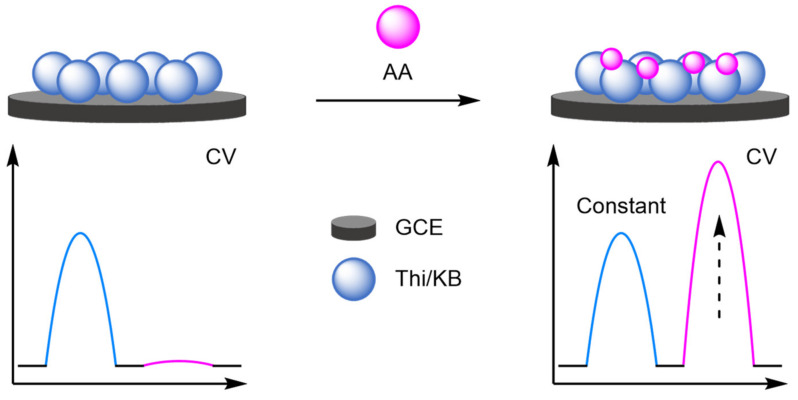
Schematic representation of Ketjen Black-based biosensor for the detection of ascorbic acid (AA).

Ketjen Black (KB) is a low-cost synthetic catalyst offering high surface area, facilitating the oxidation of electroactive compounds. Wei et al. electrodeposited a nanocomposite of KB and Thi onto a GCE (Figure 43) [134]. Thi exhibited a reversible redox potential at −220 mV, with KB catalysing the oxidation of ascorbic acid (AA) at −50 mV. The biosensor covered a dynamic range across physiological concentration of AA, with a calculated LOD of 25 µM. Song et al. incorporated an aluminium-based MOF to improve the sensitivity and selectivity for AA. Encapsulation of KB and Thi into the MOFs improved the reliability of the assay, with a correlation coefficient of 0.998 over a dynamic range of 1.41 µM–5.5 mM, with a calculated LOD of 4.6 µM. The introduction of the MOF reduced the interference from other molecules, vastly improving the reproducibility with an RSD of 3.9% over five electrodes. KB has also been used in aptasensors, where aptamer binding enhances selectivity, and KB amplifies the signal output (Figure 44) [135].

#### 2.2.5. Summary

The modification of electrodes offers a different avenue for the detection of small molecule electroactive analytes, where their facile preparation compared to DNA-modified electrodes helps to reduce costs. However, there is a trade-off with a reduction in sensitivity and selectivity, and the biosensors are limited to electroactive compounds. Methods to improve selectivity, either through biological architecture or specific recognition sites, have been developed. However, DNA labelling is still more selective. Oxidation catalysts offer a new strategy to improve biosensor sensitivity, but the limited analyte selection requires development. In general, more intensive reliability and reproducibility studies are necessary to facilitate their incorporation into point-of-use devices.

### 2.3. Unmodified Electrodes

**Figure 45:**
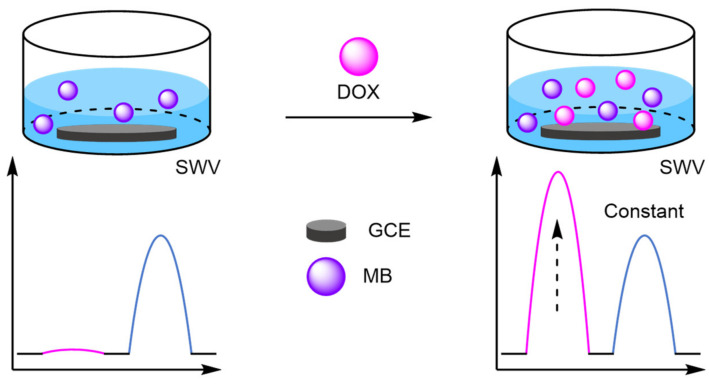
Schematic representation of an unmodified electrode for the detection of doxorubicin (DOX).

The synthetic processes required in the construction of biosensors remains a non-trivial challenge, with the immobilisation of electroactive substrates onto electrode surfaces difficult. With each new analyte, a novel strategy is required. This increases cost, labour time and requires thorough investigation. Therefore, the use of unmodified electrodes is desirable, with the use of an electrolyte solution containing a reference molecule. A general strategy was developed by Gui and Wang who used an unmodified GCE, in combination with an MB-doped electrolyte solution (Figure 45) [136]. Detection of doxorubicin (DOX) via SWV was possible with separate peaks at −270 mV (*I_MB_*) and −600 mV (*I_DOX_*). Ratiometric analysis was possible over a dynamic range of 0.01–3 µM with reliability demonstrated by the correlation coefficient of 0.991. A calculated LOD of 0.4 nM was achieved, and the signals demonstrated good thermal stability. The reproducibility of the electrodes was explored, with RSD values varying from 1.96% to 3.86% over six repeats at multiple concentrations. The authors proposed that the substitution of MB with alternative electrochemical references was feasible and went onto show that Fc [137], and Thi were also suitable as an internal reference [138].

**Figure 46:**
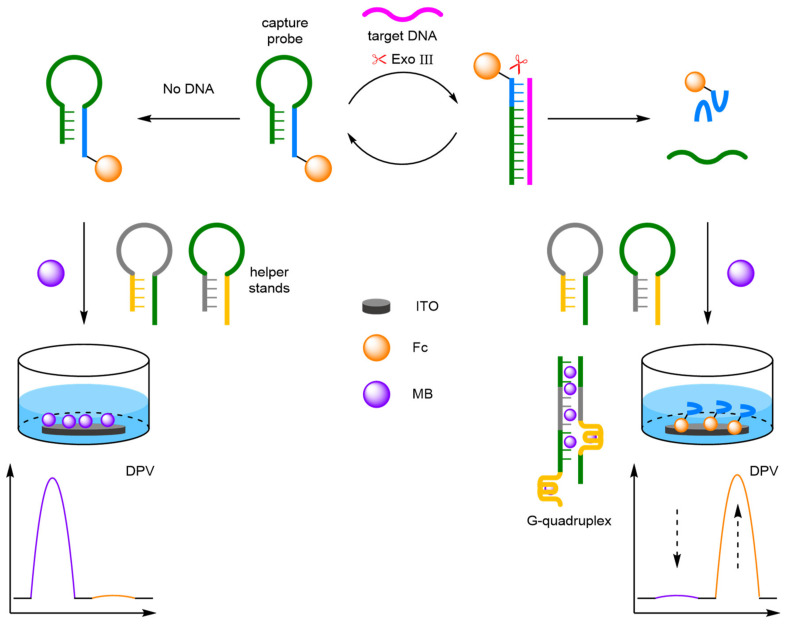
Schematic representation of diffusivity and intercalation-based unmodified electrode.

Li et al. adapted DNA-labelling methodologies in a strategy that exploited the difference in diffusivity of dNTPs towards an unmodified indium tin oxide (ITO) electrode, compared to single-stranded DNA molecules (Figure 46) [139]. A Fc-labelled capture probe in the absence of target DNA remained distal to the electrode, with a low Fc signal observed. On target DNA binding, hybridisation forms a duplex that can then be digested by an exonuclease, releasing Fc-labelled dNTPs which can then diffuse to the electrode surface, and a shortened DNA strand. The shortened strand templates G-quadruplex formation with two helper strands in an HCR. A MB-doped electrolytic solution provided a secondary signal, which in the negative reaction diffuses to the electrode surface and is intercalated into the G-quadruplex in the positive reaction. The increase in Fc signal and decrease in MB signal can then be used to monitor target DNA concentration ratiometrically. This was observed over a dynamic range of 0.01–10 pM, with a calculated LOD of 0.01 pM, comparable to alternative methods. The assay displayed a high reliability, with a correlation coefficient of 0.994.

Unmodified electrodes offer a further alternative for the development of new biosensors, where their cost-effective construction is a key factor. So far, their adoption has been limited, evidenced by the relatively small number of publications on the topic. However, with further exploration, they have potential for incorporation into point-of-use devices.

## 3. Chemodosimeters

A smaller subsection of ratiometric electrochemical sensing has been the development of chemodosimeters, a class of small molecules which offer significant differences to previous methodologies. Chemodosimeters utilise the reactivity of the target substrates to cause a trigger event, chemically altering the sensors and resulting in the irreversible conversion to a second molecule. The signal transduction process can be monitored electrochemically, with the substrate having a distinct E_ox_ compared to the released molecule. As they do not require modified electrodes and inexpensive synthetic routes, chemodosimeters trade-off a reduction in sensitivity with a corresponding cost reduction. Their simplified methodology is optimal for incorporation into point-of-use devices, making chemodosimeters an attractive target for cost-effective biosensors.

**Figure 47:**
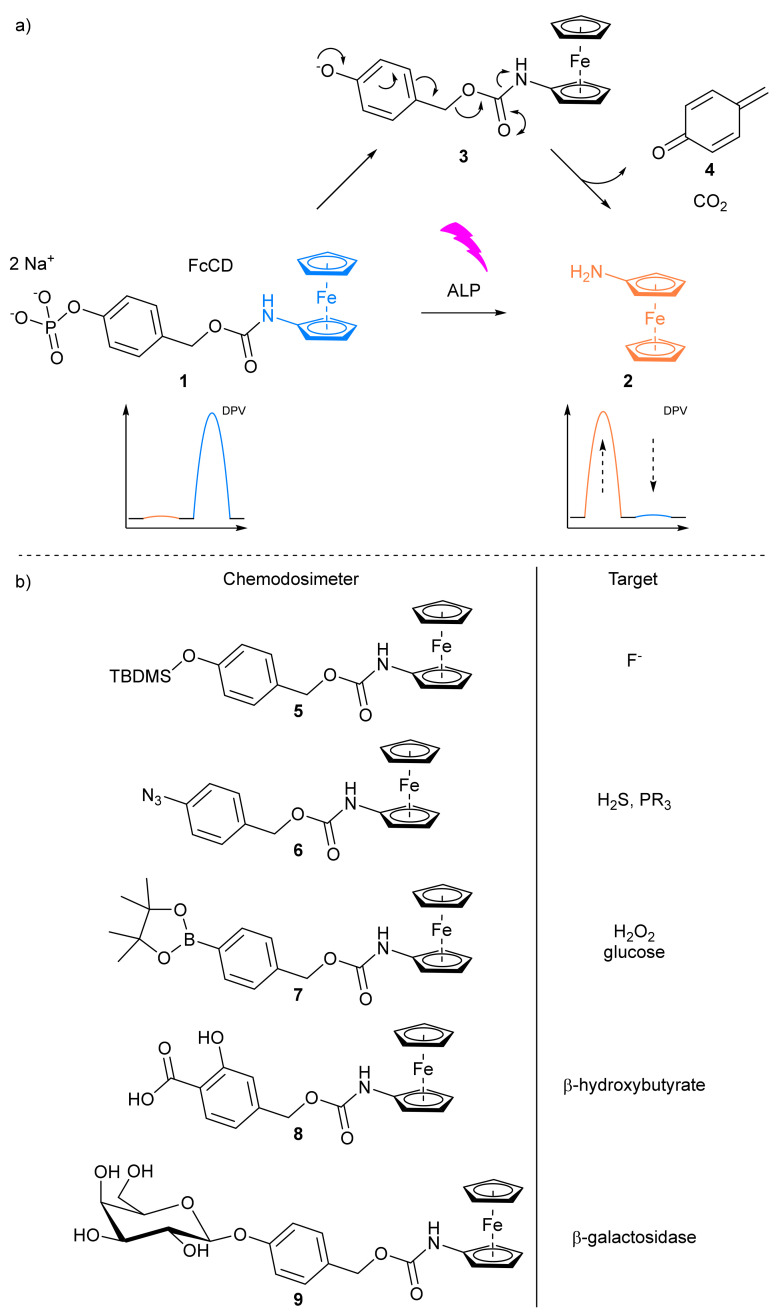
(**a**) Schematic representation of the ferrocene-based chemodosimeters for the detection of alkaline phosphatase (ALP); (**b**) selection of chemodosimeters and their targets.

The design of chemodosimeters have taken inspiration from classical prodrug strategies, where a trigger moiety is separated from the released molecule through a linker unit. A concept that has been widely incorporated into fluorescence chemodosimeters [140,141,142]. Electrochemical chemodosimeters predominantly utilise aminoferrocene **2** as the effector molecule which possesses a lower E_ox_ at −100 mV compared to 4-aminophenol at 70 mV, with the lower E_ox_ preferred to prevent the oxidation of other species. Initial electrochemical chemodosimeter design built upon the work of the Shabat group who pioneered the field with the development of an aldolase-specific chemodosimeter [143]. The E_ox_ of the ferrocene-based carbamate derivative (FcCD) occurred at 100 mV a significant separation from the peak of **2** at −100 mV. However, ratiometric analysis was not conducted with fixed potential measurement at −30 mV used instead. Frost et al. were the first group to utilise concurrent decrease in FcCD signal, with the increase in FcNH_2_ signal (Figure 47) [144] 50. An alkaline phosphatase (ALP)-specific FcCD **1** contained a phosphate trigger unit that remained stable in the absence of ALP, but was readily cleaved in its presence to release the effector. Ratiometric analysis of the two signals was possible via DPV (*I_FcCD_* 70 mV vs. *I**_2_*** −160 mV), using disposable screen-printed electrodes. The choice of electrode was important for reducing costs, and facile incorporation into point-of-use devices. An LOD of 0.4 U mL^−1^ was reported, and the FcCD was compatible with enzyme linked-immunosorbent assays (ELISAs), allowing for the potential incorporation of the substrate into multiple sensing platforms. This strategy has proved near universal, with further enzyme substrates [145,146] small molecules [147,148,149,150], and metal ions [151].

**Figure 48:**
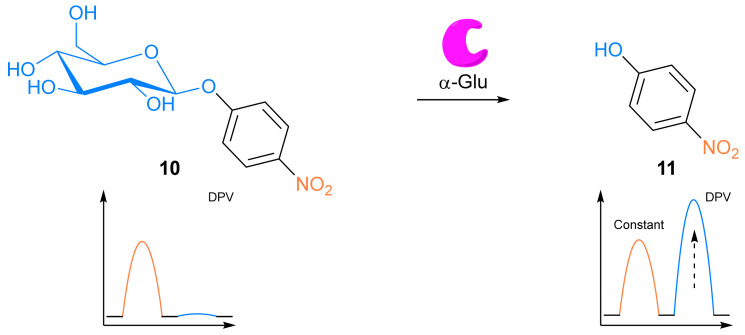
Schematic representation of a chemodosimeter for the detection of α-glucosidase (α-Glu).

A different strategy was developed by Zhao et al., where a single substrate contained two separate electroactive units [152]. One remained unaffected by the assay, supplying an internal reference, and the formation of the second moiety that could be followed electrochemically (Figure 48). The nitro- group of 4-nitrophenyl-α-d-glucopyranoside remains constant in the assay, with enzymatic cleavage of the sugar by α-glucosidase affording 4-nitrophenol. The phenol group had an E_ox_ at 50 mV and the nitro group reduced to the amine with a peak at −140 mV. The assay displayed good reliability, with a correlation coefficient of 0.994 and an LOD of 0.0056 mU mL^−1^. The reproducibility of the assay was explored with an RSD of 1.4% across ten experiments.

### 3.1. Improving Sensitivity

**Figure 49:**
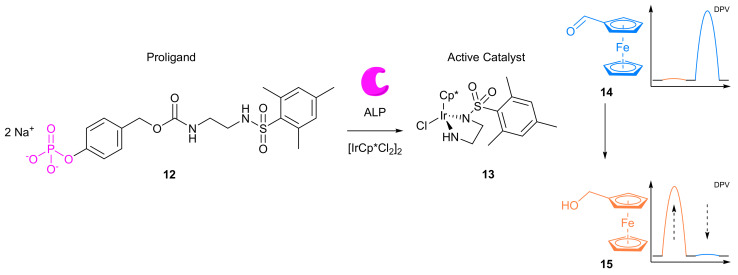
Schematic representation of a proligand-based label amplification chemodosimeter for the detection of alkaline phosphatase (ALP).

The major drawback of chemodosimeters is their reduced sensitivity for target analytes. Therefore, to overcome the issue, Frost et al. modified their ratiometric electrochemical sensor for ALP to release an amplification agent, that switched on a catalytic cycle (Figure 49) [153]. The proligand carbamate derivate contained a tosylated diamine ligand, that on releasing enhances the catalytic activity of an iridium precatalyst. The active catalyst converts ferrocene carboxaldehyde **14** to ferrocene methanol **15**, with a change in E_ox_ from 225 mV to −50 mV observed via DPV. In the absence of ALP, no ligand release would occur and there would be minimal reduction of ferrocene carboxaldehyde. The amplification strategy improved the LOD to 7.6 pM after 3 mins, highly desirable for incorporation into point-of-use devices.

### 3.2. Summary

The development of electrochemical chemodosimeters has had important implications for the biosensor field. Their design has offered feasible alternatives to optical-based assays, their simplicity is cost effective and favourable for incorporation into point-of-use devices. The adaptable design makes configuration to new targets facile. However, thorough selectivity studies are required during assay optimisation. The current range of electrochemical chemodosimeters is small in comparison to colourimetric and fluorescent equivalents, therefore, there is significant scope to develop the field further. Initial work to improve sensitivity has been conducted, though alternative strategies are required to expand current methodologies. Taking this into account, chemodosimeters are a promising research area for biosensor design. 

## 4. Dual Channel

Signal overlap in voltammograms can prevent reliable analysis often leading to false positives. Construction of biosensors must therefore consider peak positioning to minimise this overlap, which becomes more apparent in complex sampling, where non-specific interference must be overcome before accurate sensing is feasible. An elegant strategy is to separate the two signals to different electrodes preventing peak overlap. As both electrodes are subjected to the same conditions, environmental factors are accounted for, and the benefits of ratiometric analysis is maintained. One electrode is typically labelled with a specific binding moiety, with the second electrode labelled with an electrochemical reference. Voltammograms obtained for both electrodes are obtained, and then ratiometric analysis conducted to calculate analyte concentration.

### 4.1. One Reference Electrode

**Figure 50:**
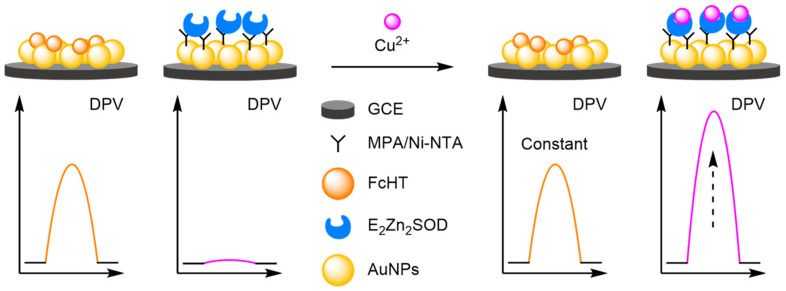
Schematic representation of a dual-channel biosensor for the detection of Cu^2+^.

Dual-channel methodology was first proposed by Tian et al. for the detection of Cu^2+^ in vivo [154]. Carbon fibre microelectrodes (CFMEs) was modified with cysteamine and AuNPs before the reference working electrode was labelled with ferrocene hexanethiol (FcHT) (Figure 50). The second working electrode was further functionalised with 3-mercaptopropionic acid, then nickel nitriloacetic acid. Finally, the recognition element, a copper-free derivative of bovine erythrocyte, copper-zinc superoxide dismutase (E_2_Zn_2_SOD) was bound through a metal chelate effect. Cu^2+^ binding to the recognition site results in a reduction peak observable via DPV at 150 mV vs. Ag|AgCl. The reference remains constant at 390 mV (*I_Fc_*), with significant overlap with the Cu^2+^ reduction peak, justifying the use of a dual-channel system. The reproducibility of the sensor was explored with 10 electrodes affording an RSD of 5.7%. An LOD of 3 nM was calculated with a dynamic range of 10 nM–35 µM, suitable for testing Cu^2+^ concentration in live rat brains. Tian et al. proved that the methodology could be readily modified to investigate pH in vivo [155]. Utilising the same reference working electrode, the second working electrode was modified with a pH sensitive pyridine-functionalised Fc moiety. The sensor had a dynamic range of pH 5.9–8.0, with a detection limit of 0.13 pH. The dual-channel approach was made suitable for repetitive in vivo measurements by the introduction of an ethylenediaminetetraacetic acid wash, regenerating the electrode in situ [156].

### 4.2. Two Working Electrodes

**Figure 51:**
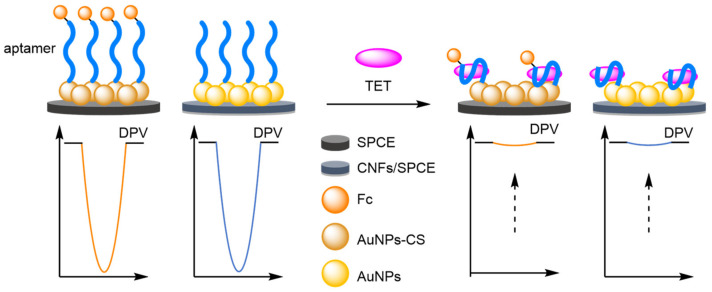
Schematic representation of a dual-channel aptasensor for the detection of tetracycline (TET).

An alternative dual-channel approach was taken by Zhang et al., who constructed two working electrodes labelled with different electrochemical labels, Fc and carbon nanofibers (CNFs), for the detection of tetracycline (TET) (Figure 51) [157]. Two separate aptasensors were constructed, the first started with a SPCE modified with AuNP-chitosan (CS) composite, to which the Fc-labelled aptamer was immobilised. The second aptasensor contained a CNF composite on the SPCE, followed by addition of AuNPs. An unlabelled aptamer was immobilised onto the AuNPs. In the presence of tetracycline, aptamer binding reduces the current observed for both electrodes. Ratiometric analysis utilising the change in currents proved a highly reliable methodology, with a reported correlation coefficient of 0.996 across a dynamic range of 10 ng L^−1^–1 µg L^−1^, and 1 µg L^−1^–mg L^−1^ for the detection of tetracycline. The reproducibility of the assay was explored with an intra- and inter-aptasensor RSD of 4.62% and 3.4%, respectively.

### 4.3. Summary

Dual-channel biosensors overcome the constant issue associated with electrochemical sensing of signal overlap. However, this comes with the requirement to develop two separate electrodes, doubling the research time. The separation of signal has distinct advantages in complex sampling situations, including in vivo, making further work into dual-channel biosensors important.

## 5. Conclusions

The vast array of biosensors design means that for each analyte, a selection of strategies is feasible, with it up to the researcher to determine which route is optimal. Several observations remain consistent, and ratiometric electrochemistry offers improved reliability and reproducibility, but there is only a minimal gain in sensitivity. An increase in sensitivity is achieved through other means, including amplification strategies or rigorous optimisation. The reproducibility of the biosensors must be explored, otherwise their suitability is not fully understood. Secondly, high selectivity is possible through the correct choice of recognition element, although stringent screening is necessary to confirm the desired selectivity. Finally, stability studies are important, without which incorporation into point-of-use devices is difficult. When constructing a biosensor, these factors should be at the forefront. It is needless to overengineer an assay if a simpler, cheaper equivalent is suitable. In general, DNA-based biosensors offer excellent sensitivity and selectivity, though modified electrodes offer a cheaper alternative for electroactive analytes, and dual-channel biosensors are designed for complex sampling. Chemodosimeters are highly desirable for point-of-use incorporation, but the reduction in sensitivity may not be favourable. Therefore, it is only through sensible and rational design and strict testing that biosensors can be transitioned from the lab and into the field.
